# Whole-genome sequencing reveals the genetic mechanisms of domestication in classical inbred mice

**DOI:** 10.1186/s13059-022-02772-1

**Published:** 2022-09-26

**Authors:** Ming Liu, Caixia Yu, Zhichao Zhang, Mingjing Song, Xiuping Sun, Jaroslav Piálek, Jens Jacob, Jiqi Lu, Lin Cong, Hongmao Zhang, Yong Wang, Guoliang Li, Zhiyong Feng, Zhenglin Du, Meng Wang, Xinru Wan, Dawei Wang, Yan-Ling Wang, Hongjun Li, Zuoxin Wang, Bing Zhang, Zhibin Zhang

**Affiliations:** 1 State Key Laboratory of Integrated Management of Pest Insects and Rodents, Institute of Zoology, Chinese Academy of Sciences, Beijing, China; 2 International Society of Zoological Sciences, Beijing, China; 3 State Key Laboratory of Stem Cell and Reproductive Biology, Institute of Zoology, Chinese Academy of Sciences, Beijing, China; 4 Beijing Institute of Genomics, Chinese Academy of Sciences and China National Center for Bioinformation, Beijing, China; 5 National Genomics Data Center, Beijing Institute of Genomics, Chinese Academy of Sciences, Beijing, China; 6 Novogene Bioinformatics Institute, Beijing, China; 7 Glbizzia Biosciences, Beijing, China; 8 Institute of Laboratory Animal Science, Chinese Academy of Medical Sciences, Beijing, China; 9 House Mouse Group, Research Facility Studenec, Institute of Vertebrate Biology of the Czech Academy of Sciences, Brno, Czech Republic; 10 Julius Kühn-Institute, Federal Research Centre for Cultivated Plants, Institute for Plant Protection in Horticulture and Forests / Institute for Epidemiology and Pathogen Diagnostics, Münster, Germany; 11 School of Life Science, Zhengzhou University, Zhengzhou, Henan China; 12 Institute of Plant Protection, Heilongjiang Academy of Agricultural Sciences, Harbin, Heilongjiang China; 13 School of Life Sciences, Central China Normal University, Wuhan, Hubei China; 14 Institute of Subtropical Agriculture, Chinese Academy of Sciences, Changsha, Hunan China; 15 CAS Center for Excellence in Biotic Interactions, University of Chinese Academy of Sciences, Beijing, China; 16 Plant Protection Research Institute Guangdong Academy of Agricultural Sciences, Guangzhou, Guangdong China; 17 Institute of Plant Protection, Chinese Academy of Agricultural Sciences, Beijing, China; 18 Department of Psychology and Program in Neuroscience, Florida State University, Tallahassee, FL 32306 USA

**Keywords:** *Mus musculus*, Domestication, Positively selected gene, Genome sequencing, Alternative splicing

## Abstract

**Background:**

The laboratory mouse was domesticated from the wild house mouse. Understanding the genetics underlying domestication in laboratory mice, especially in the widely used classical inbred mice, is vital for studies using mouse models. However, the genetic mechanism of laboratory mouse domestication remains unknown due to lack of adequate genomic sequences of wild mice.

**Results:**

We analyze the genetic relationships by whole-genome resequencing of 36 wild mice and 36 inbred strains. All classical inbred mice cluster together distinctly from wild and wild-derived inbred mice. Using nucleotide diversity analysis, Fst, and XP-CLR, we identify 339 positively selected genes that are closely associated with nervous system function. Approximately one third of these positively selected genes are highly expressed in brain tissues, and genetic mouse models of 125 genes in the positively selected genes exhibit abnormal behavioral or nervous system phenotypes. These positively selected genes show a higher ratio of differential expression between wild and classical inbred mice compared with all genes, especially in the hippocampus and frontal lobe. Using a mutant mouse model, we find that the SNP rs27900929 (T>C) in gene *Astn2* significantly reduces the tameness of mice and modifies the ratio of the two *Astn2 (a/b)* isoforms.

**Conclusion:**

Our study indicates that classical inbred mice experienced high selection pressure during domestication under laboratory conditions. The analysis shows the positively selected genes are closely associated with behavior and the nervous system in mice. Tameness may be related to the *Astn2* mutation and regulated by the ratio of the two *Astn2 (a/b)* isoforms.

**Supplementary Information:**

The online version contains supplementary material available at 10.1186/s13059-022-02772-1.

## Background

Animal domestication is a special evolutionary event under artificial selection accompanied with the history of human society. Over the course of domestication by humans, animals are forced to adapt to new environments and exhibit characteristics distinct from their wild relatives, such as changes of coat color, more frequent estrus cycles, and increased tameness [1]. Domesticated animals can be divided into three types: farm animals (like pigs and chickens), pet animals (like cats and dogs), and experimental animals (like mice and rats) [2]. In farm and pet animals, it has been demonstrated that a number of genes/loci were relevant to traits for animal production, such as body size, fur color, immune system, and reproduction [3, 4]. In mice and rats, increased tameness is considered the critical trait of domestication [2, 5]. Modification in behavior, especially increased tameness, occurs in nearly all domestic animals [1, 6]. Loci associated with the nervous system and/or behavior are observed in dogs and cats [7, 8], as well as in pigs, chickens, sheep, and goats [9–12]. Recently, genes related to the nervous system have been shown to be involved in the domestication of rats [13].

Laboratory inbred mice strains are used world-wide as animal models and can be classified into two groups: wild-derived inbred mice and classical inbred mice [14]. Classical inbred mice that were developed from fancy mice are artificial hybrids with mixed genomes of *Mus musculus domesticus* (*M. m. domesticus*), *M. m. musculus*, and *M. m. castaneus*. Genome-wide studies based on wild-derived inbred mice [15–18] reveal that *M. m. domesticus* is the predominant source of the classical inbred mice, contributing 80–95% of the genome of classical inbred mice, with another 5–10% originating from *M. m. musculus*, and less than 4% from *M. m. castaneus* [15, 16, 18]. Fancy mice were severely inbred and kept as pet animals [14]. Coadaptation to the laboratory life and continual manipulation by humans suggests that the classical inbred mouse strains should be highly selected for various domestic traits. Wild-derived inbred mice usually originate from a group of local wild individuals as genetic models, with the trait and genetic backgrounds more similar to wild mice than classical inbred mice [19]. Several studies demonstrate that classical inbred strains show higher tameness than wild-derived inbred strains [2, 20, 21], and variation in neural and endocrine systems are also apparent [22, 23]. However, mechanisms of laboratory domestication and relevant selected genes of mice are not fully understood. Considering the mixed genomes of classical inbred mice, it is important to trace the developments of the domestication of mice using genome resequencing data from a large population of wild house mice.

This study aims to analyze genetic mechanisms of domestication of mice. To account for the mixed genetic background of inbred mice, we resequenced genomes of 36 wild mice with 10× depth on average from *M. m. domesticus*, *M. m. musculus*, and *M. m. castaneus*, separately. By comparing whole genomes of 36 inbred mouse strains downloaded from the Sanger Institute, we identified positive selected genes (PSGs), examined their expression via RNA-seq, and tested the function of some selected loci in this model organism.

## Results

### Samples and whole-genome sequencing

To identify the genomic selection of domestication in classical inbred mice, we obtained samples from 36 wild mice for genome sequencing, including 11 samples of *M. m. domesticus*, 9 samples of *M. m. musculus*, and 16 samples of *M. m. castaneus* (Additional file 1: Fig. S1 and Table S1). The genome sequences of 36 inbred laboratory mice were downloaded from the Sanger Institute website [24] and include 29 classical inbred strains and 7 wild-derived inbred strains (Additional file 1: Fig. S1 and Table S2).

The whole-genome resequencing data was generated for the samples from 36 wild mice by Illumina technology. Approximately 1.4 Tb data was acquired. Raw sequencing data for the 36 wild mice ranged from 24.4 to 54.3 Gb (Additional file 1: Table S3). After mapping to the mouse reference genome (GRCm38.p2; accessed at https://www.ncbi.nlm.nih.gov/assembly/GCF_000001635.22/) using BWA [25], we obtained sequencing depths of the 36 wild mice, which ranged from 9.0× to 20.7×, and the genome coverage ranged from 91.7 to 95.4% (Additional file 1: Table S3).

### Genomic variation in mice

The whole-genome resequencing data yielded 17,295,344 SNPs across the 11 wild *M. m. domesticus* samples (Additional file 1: Table S4). Totally, 143,421 SNPs were distributed in exons, 3,997,285 in introns, and 9,949,066 in intergenic regions. The genome resequencing analysis from 9 wild *M. m. musculus* samples provided 29,740,023 SNPs (Additional file 1: Table S4), of these 227,146 SNPs in exons, 6,989,778 in introns, and 17,042,733 in intergenic regions. The 16 *M. m. castaneus* generated 38,325,000 SNPs: 269,586 SNPs were in exons, 9,045,198 in introns, and 21,931,641 in intergenic regions (Additional file 1: Table S4). In contrast, the 29 classical inbred mice included 100,276 SNPs in exons, 3,130,111 in introns, and 7,051,985 in intergenic regions (Additional file 1: Table S4).

The number of SNPs varied among the 6 wild-derived inbred mice strains originated from *M. musculus* (Additional file 1: Table S5). The 3 wild-derived inbred strains from *M. m. domesticus* (WSB/EiJ, LEWES/EiJ, and ZALENDE/EiJ) exhibited a similar number of SNPs (4,884,269, 4,903,673, and 5,603,599, respectively) per strain (Additional file 1: Table S5-S6). The other 3 wild-derived inbred mice strains CAST/EiJ, PWK/Ph, and MOLF/EiJ exhibited a higher number of SNPs (15,091,063, 14,757,431, and 14,203,889, respectively) than the 3 *M. m. domesticus* wild-derived inbred mice (Additional file 1: Table S5-S6).

### Phylogenetic analysis of mice

**Fig. 1:**
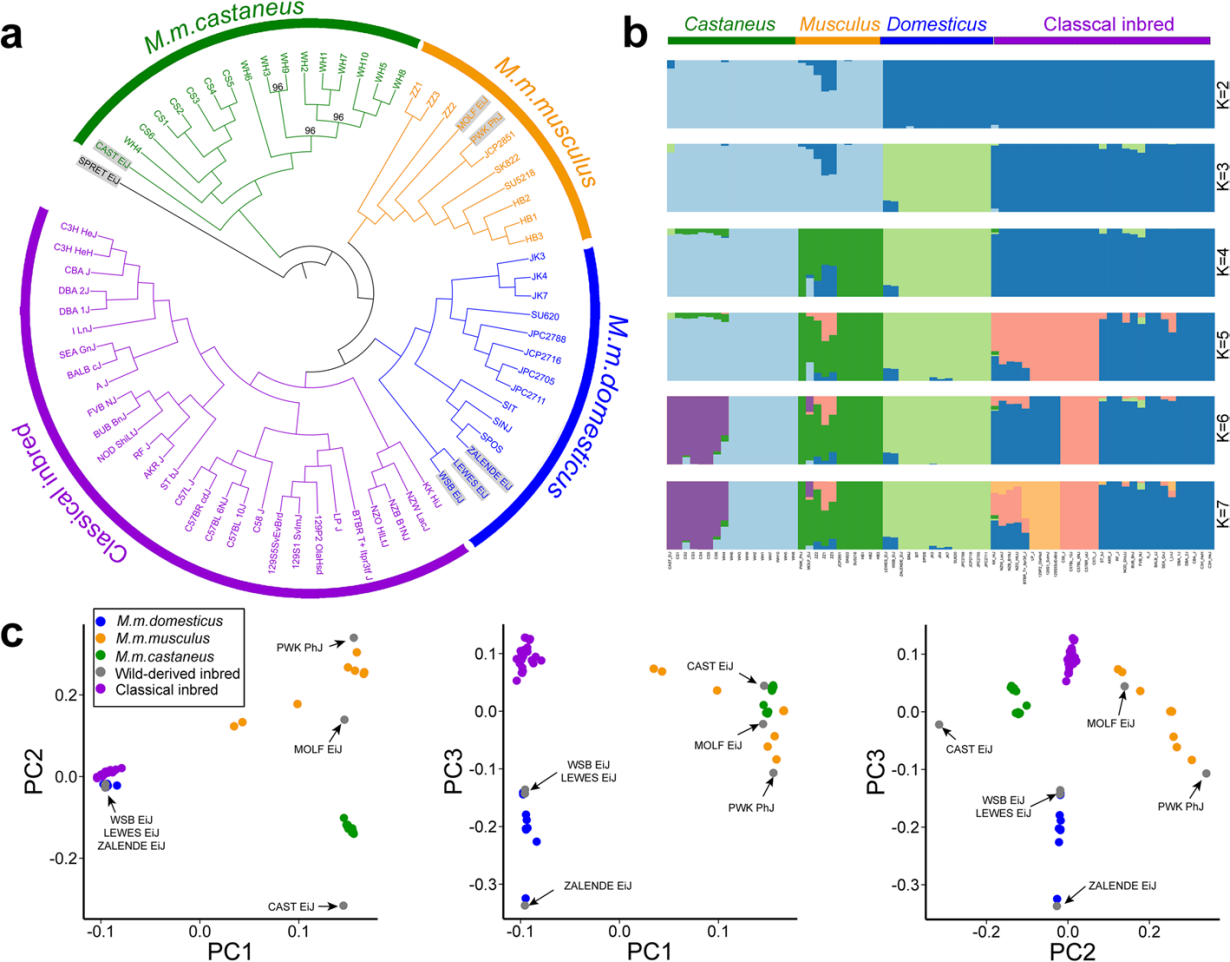
Phylogenetic relationships and population structure of mice. **a** Phylogenetic tree of mice. Wild *M. m. domesticus* (blue), *M. m. musculus* (yellow), and *M. m. castaneus* (green) are clustered with their wild-derived inbred mice relatives (with gray background) and separated from classical inbred mice (purple). SPRET/EiJ representing *M. spretus* is set as outgroup. All the bootstrap values are 100 except for the 3 bootstrap values marked in the panel. **b** Population structure analysis of the mice by ADMIXTURE. CV errors are low when *K* = 4 to 7 (see Additional file 1: Fig. S2). Wild *M. m. domesticus*, *M. m. musculus*, *M. m. castaneus* and classical inbred mice are clustered from each other when *K* = 4. **c** Principal component analysis (PCA) of wild mice, wild-derived mice, and classical inbred mice. The components with the percentage of eigenvalue > 5% (PC1–PC3, see Additional file 1: Fig. S3) are included. The strain names of the wild-derived strains are marked by arrows

To assess the relationships among classical or wild-derived inbred mice and wild mice, we performed a phylogenetic analysis based on 26,376,666 SNPs (Fig. 1a). In the resulting neighbor joining tree (Fig. 1a), all 71 mice are clustered into four groups from the outgroup SPRET/EiJ. The first group is composed of 16 wild *M. m. castaneus* and wild-derived inbred strain CAST/EiJ, originating from *M. m. castaneus* (Additional file 1: Table S6). The second group contains 9 wild *M. m. musculus* and wild-derived inbred strains PWK/PhJ and MOLF/EiJ, which originate from *M. m. musculus* and *M. m. molossinus*, respectively (Additional file 1: Table S6). The third group includes 11 wild *M. m. domesticus* and 3 wild-derived inbred strains WSB/EiJ, LEWES/EiJ, and ZALENDE/EiJ, which originate from *M. m. domesticus* (Additional file 1: Table S6). The fourth group is composed of 29 classical inbred strains. Hence, wild individuals from the *M. musculus* subspecies and their wild-derived relatives are clustered distinctly from each other. The classical inbred mouse strains are found in a separate group suggesting the founder effects when a limited number of progenitors were used to derive classical inbred stains, or the admixture of the 3 subspecies (mainly *M. m. domesticus*), or the presence of artificial selection during mouse domestication, or a combination of all factors. The clustering of classical inbred strains with the wild *M. m. domesticus* is consistent with the view that the classical inbred mice predominately originate from this subspecies [15, 16, 18]. The relationships from phylogenetic analyses are also supported by Bayesian clustering analysis using ADMIXTURE [26] (Fig. 1b and Additional file 1: Fig. S2) and principal component analysis (PCA) [27] (Fig. 1c and Additional file 1: Fig. S3).

### Identification of positively selected genes (PSGs)

**Fig. 2:**
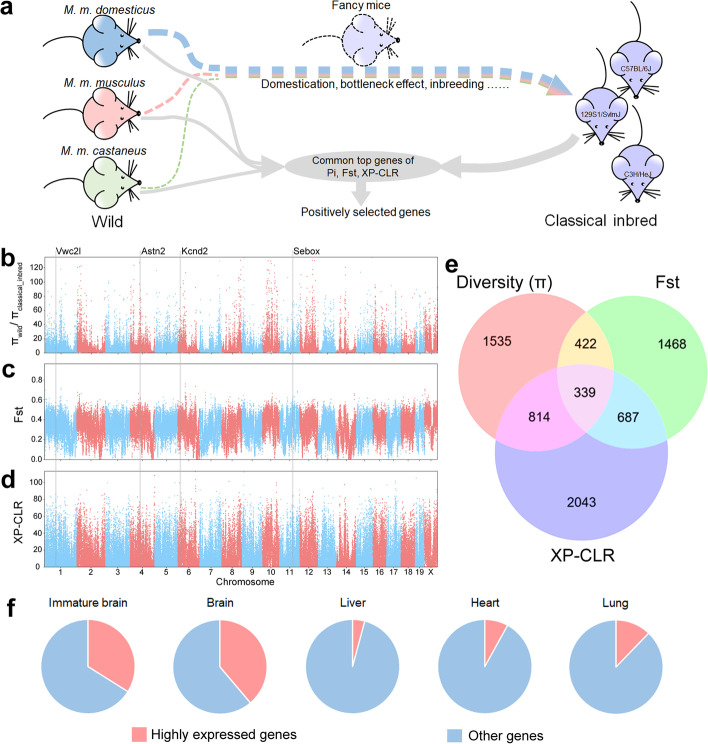
Positively selected genes (PSGs) associated with domestication in mice. **a** The diagram of domestication history of classical inbred mice and the strategy for selecting PSGs. **b–d** Manhattan plots indicate high and low positive selected regions by nucleotide diversity (π_wild_/π_classical_inbred_), Fst, and XP-CLR. The highly selected genes marked by gray lines are individually validated by the following experiments. **e** The Venn diagram depicting genes identified by three independent approaches illustrated in **b–d**. The intersection includes finally selected 339 PSGs. **f** The ratios of highly expressed genes in the 339 PSGs are disproportionally enriched in brain. “Immature brain” indicates all the average RPKM of embryonic central neuro system tissues aged 11.5, 14, and 18 days *post coitum*; “Brain” indicates all the average RPKM of the adult brain tissues cerebellum, cortex, and frontal lobe

As we noted, the genetic background of classical inbred mice is a mosaic of the 3 mouse subspecies [16, 18]; consequently, genomes representing all subspecies should be used for identifying the positively selected genes (PSGs) presumably associated with domestication in classical inbred mice strains. Classical inbred mice experienced a high degree of inbreeding, severe population bottlenecks (founder effect), and genetic drift in its history of domestication [28], which significantly decreased genetic diversity. Genetic drift or founder effect randomly brings sharp allele frequency variation in a number of sites, which were mixed with the selected sites and hard to distinguish. Multiple and independent approaches should be used to alleviate the disturbance of these accidental factors. Based on the phylogenetic analysis (Fig. 1) that separated classical inbred mice from their wild relatives, we assumed that genetic differences separating classical inbred mice could disclose genetic features of domestication. Hence, to explore the genetic mechanisms underlying the domestication of mice, we assigned all the classical inbred strains as the “classical_inbred group” and wild mouse individuals and wild-derived inbred strains that originate from *M. musculus* as the “wild group” (Figs. 1 and 2a). The genomes of the two groups were scanned using three independent approaches: nucleotide diversity (*π*_wild_/*π*_clsssical_inbred_), Fst [29], and XP-CLR [30] (Fig. 2b–d), which helped to alleviate the disturbance of the founder effect. The top 5% ranked genes of each strategy were selected, and the intersection of the genes was considered as PSGs associated with laboratory mouse domestication.

### PSGs closely related to the function of the nervous system

To detect the nucleotide diversity (*π*_wild_/*π*_classsical_inbred_) variations across the genome, we scanned the genome with windows of 40 kb and step size of 20 kb (Fig. 2b). The top 5% ranked windows of nucleotide diversity contain 3110 genes (Additional file 2: Table S7). Gene ontology (GO) analysis revealed that these genes were mostly enriched in the function of the nervous system (Additional file 1: Fig. S4 and Additional file 2: Table S8), including “positive regulation of neuron differentiation” (GO:0045666) and “neuron to neuron synapse” (GO:0098984). Scanning the genomes with Fst analysis (Fig. 2c) revealed 2916 genes in top 5% ranked windows (Additional file 2: Table S9). Gene ontology analysis indicated that genes associated with the nervous system were still enriched (Additional file 1: Fig. S5 and Additional file 2: Table S10), like “synapse organization” (GO:0050808) and “neuron to neuron synapse” (GO:0098984). XP-CLR detected 3883 genes enrolled in the top 5% ranked windows (Fig. 2d and Additional file 2: Table S11). Gene ontology analysis revealed the enriched categories (Additional file 1: Fig. S6 and Additional file 2: Table S12) such as “regulation of membrane potential” (GO:0042391) and “cell-cell junction” (GO:0005911), which are associated with the function of nervous system.

To narrow the list of selected genes of domestication, the common top 5% ranked genes acquired from the three independent approaches (Fig. 2b–d) were merged and the 339 PSGs that intersected were listed (Fig. 2e and Additional file 2: Table S13). Gene ontology analysis (Additional file 1: Fig. S7 and Additional file 2: Table S14) showed that “regulation of membrane potential” (18 genes, GO:0042391), “synapse organization” (14 genes, GO:0050808), “transporter complex” (13 genes, GO:1990351), and “GABA-ergic synapse” (9 genes, GO:0098982) were the top categories (Additional file 1: Fig. S7 and Additional file 2: Table S14), indicating that the neuro-associated functions play an important role in the domestication of mice. Using the same strategy, we analyzed the selected genes using only the classical inbred mice and the 36 wild mice (i.e., excluding the six wild-derived inbred strains). There were 355 selected genes (Additional file 2: Table S15), and these were very similar to the list of 339 PSGs.

We searched for expression profiles of selected genes in the Mouse ENCODE transcriptome database (PRJNA66167) [31], in which there are 286 genes of the 339 PSGs recorded. Among the 286 genes, 97 were highly expressed (at least 2-fold of the average RPKM, reads per kilobase per million mapped) in the immature brain (33.9%) and 111 were highly expressed in the brain (38.8%), while the highly expressed gene number in other tissues and organs was only 25.3 (8.9%) on average (Fig. 2f and Additional file 1: Fig. S8). In the liver, heart, lung, and kidney, the number of highly expressed gene was 12 (4.2%), 23 (8.0%), 35 (12.2%), and 20 (7.0%), respectively (Fig. 2f and Additional file 1: Fig. S8). We also performed a rank-sum test between immature brain/brain and other tissues and found 140 of the 286 genes showed significantly higher expression in immature brain/brain than in other tissues (Additional file 2: Table S16). The common positively selected genes exhibit a close and special relation to the central nervous system, again indicating that behaviorally associated modifications make up core changes in mice domestication.

We further explored the database of mutant or knockout mice that directly link phenotypes to gene function [32]. Searching the database of mutant or knockout mouse models (conditional or conventional knockout, chemical induced, or spontaneous mutation) accessed at the Mouse Genome Informatics website (http://www.informatics.jax.org) [32], revealed that of the 339 PSGs, 245 genes have mutant or knockout mouse models and 125 of the models (51.0%, *n* = 245) have phenotypes associated with abnormality in behavior and/or the nervous system (Additional file 2: Table S17), approximately 1.7 fold of total genes in the database (30.1%, *n* = 14743, Additional file 1: Fig. S9), indicating the core role of behavioral modification in mice domestication. Worthy of mention is that some PSGs without known behavioral phenotypes are associated with human mental illnesses, e.g., *CBLN4* (Alzheimer’s disease [33]), *WBSCR17* (Parkinson’s disease [34, 35], autism [36]), and *ASTN2* (autism [37–42], Alzheimer’s disease [43, 44], intellectual disability [45, 46], schizophrenia [47, 48], and attention deficit/hyperactivity disorder [40, 49–51]).

### PSGs exhibit highly enriched differentially expressed genes numbers in brain tissues of wild and classical inbred mice

**Fig. 3:**
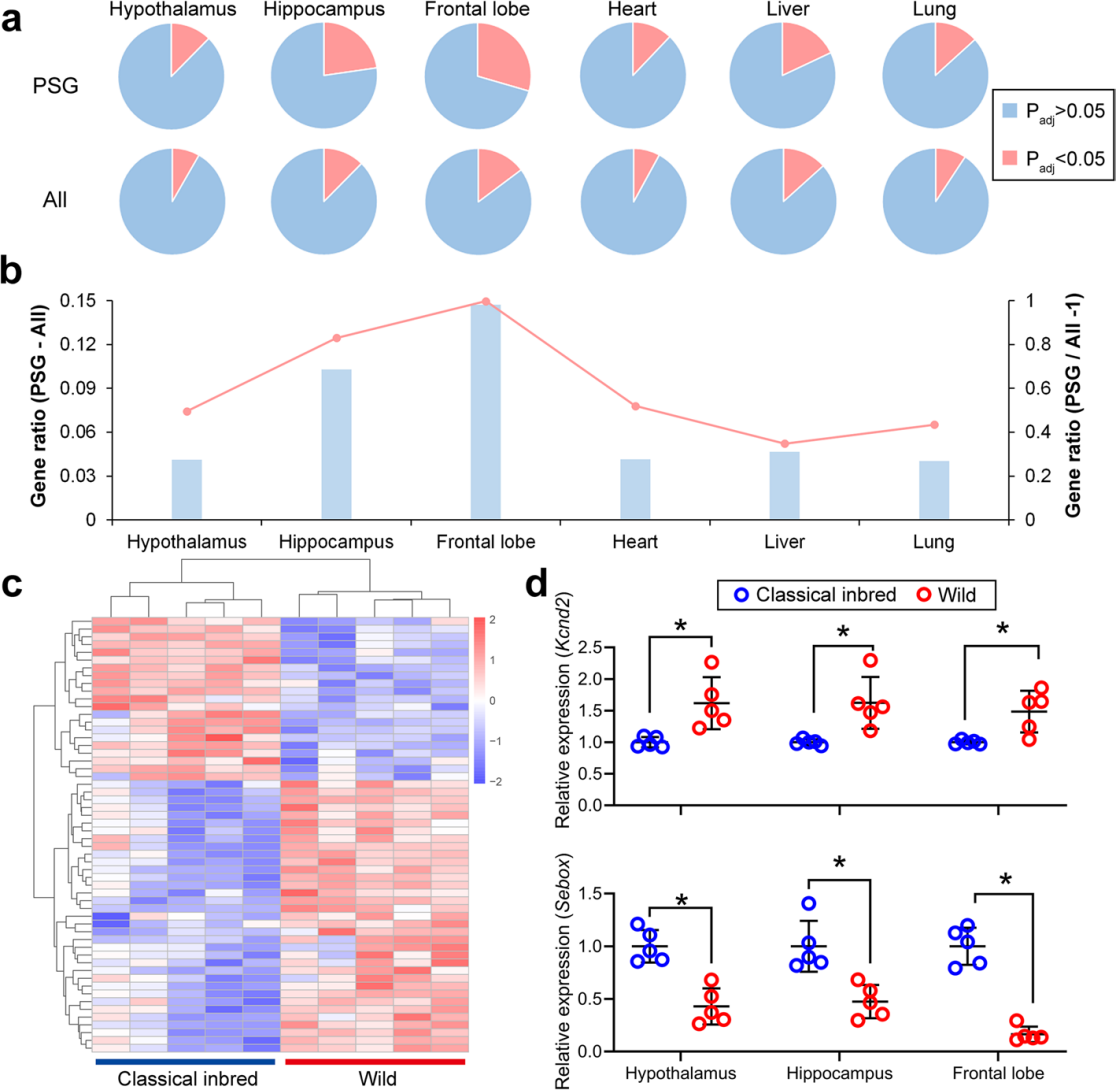
Proportion of differently expressed genes increased in the 339 PSGs. **a** Ratio of differently expressed genes in the hypothalamus, hippocampus, frontal lobe, heart, liver, and lung. Genes with *p* adjustment < 0.05 and FPKM > 1 are considered differently expressed. **b** Elevation of differently expressed gene ratio in the 339 PSGs compared with all genes. The left vertical axis corresponds to the columns, indicating the increased part of differently expressed gene ratio in the 339 PSGs compared with all genes. The right vertical axis corresponds to the dot and line, indicating the increased percentages of differently expressed gene ratio in the 339 PSGs compared with all genes. **c** The heatmap indicates the expression changes of the 56 common differently expressed genes (merged from hippocampus and frontal lobe) in the hippocampus. **d** The qPCR validation for the gene expression of *Kcnd2* (upper panel) and *Sebox* (bottom panel) between classical inbred (*n* = 5 in each group) and wild (*n* = 5 in each group) mice. Each circle indicates one individual mouse, and error bars are standard error of mean (SEM). * indicates *p* < 0.05

To explore whether the PSGs are associated with nervous system, RNA sequencing was performed between classical inbred mice (C57BL/6J) and wild mice (wild-captured mice) in six types of tissue, including three tissues from the brain: hypothalamus, hippocampus, and frontal lobe; as well as three non-brain tissues: heart, liver, and lung. The ratio of differentially expressed genes between wild mice and classical inbred strains increased in all the six types of tissues (Fig. 3a, b). In the hypothalamus, heart, liver, and lung, the increased ratio of differentially expressed genes was approximately 4% (1.3-fold) in PSGs as compared with all genes, while in the hippocampus and frontal lobe, the increased ratio of differentially expressed genes reached as high as approximately 10% (1.8-fold) and 15% (2.0-fold), respectively (Fig. 3a, b, Additional file 2: Table S18-S19). This result was consistent with findings in rats [13], suggesting that gene expression in hippocampus and frontal lobe may be closely associated to modifications in nervous system function in adult mice. The hypothalamus is an important brain region for behavioral performance in mice, but the result of RNA-seq in adult mice did not show a higher ratio of differentially expressed genes as compared to the three non-brain tissues (Additional file 2: Table S20). Hence, the common differentially expressed genes between the hippocampus and frontal lobe were used to select the final differentially expressed genes in brains.

In PSGs, there are 56 common differentially expressed genes between the hippocampus and frontal lobe (Fig. 3c and Additional file 1: Fig. S10). A subset of these genes shows different and clear variations in the following qPCR validation (Figs. 2b–d and 3d). The *Kcnd2* gene is a gene encoding the voltage-gated potassium channel, of which the expression is approximately 50% higher in the brain tissue of wild mice as compared with classical inbred mice (Fig. 3d, upper panel). *Kcnd2* belongs to the GO ontologies “regulation of membrane potential,” “positive regulation of ion transport,” “transporter complex,” and “GABA-ergic synapse,” which are highly enriched by PSGs and associated with the nervous system and behavior in this study (Additional file 1: Fig. S7 and Additional file 2: Table S14). It has been shown that *Kcnd2* is essential in the regulation of synaptic plasticity [52, 53], and knockout mice exhibit enhanced sensitivity to mechanical stimuli [54]. Mutations of *KCND2* in humans are related to autism [54, 55]. All this evidence suggests that *Kcnd2* is essential for the function of the nervous system and is involved in changes in behavior as a result of the domestication of mice. Another differentially expressed gene, *Sebox*, exhibited more than a 50% decrease in the brains of wild mice as compared with those of classical inbred mice (Fig. 3d, bottom panel). Unlike *Kcnd2*, studies of *Sebox* are rare but one study reported that this gene is most highly expressed in the adult brains of mice [56], suggesting this gene may be involved in the nervous system and changes in behavior during the domestication process.

Some genes shown to be only differentially expressed in the hippocampus or frontal lobe by RNA-seq could also be validated by qPCR (Fig. 2b–d and Additional file 1: Fig. S11). The *Vwc2* gene is mainly expressed in the brain and it has been suggested that it plays a role in the domestication of dogs [7]. In mice, the expression of *Vwc2* is very low, but a gene named *Vwc2l* with a similar structure and a much higher expression level is significantly and highly expressed in the hypothalamus and frontal lobe of wild mice (Additional file 1: Fig. S11). The results suggested that *Vwc2l* may be involved during behavioral selection in mice domestication.

### Positive selective locus *Asnt2* alters tameness in mice

**Fig. 4:**
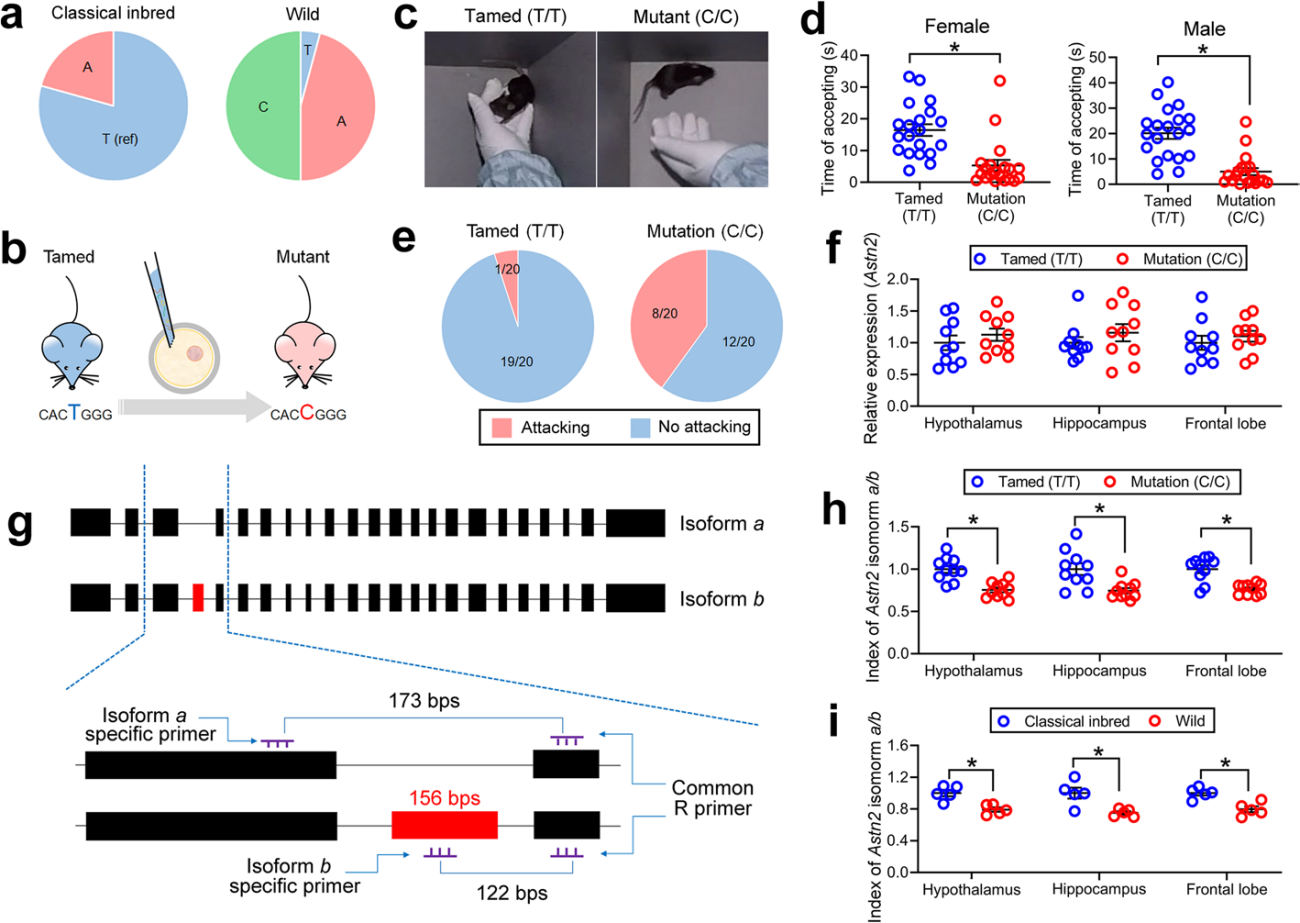
Effects of SNP rs27900929 located at *Astn2* intron on mouse tameness. **a** Allele frequency of SNP rs27900929 in classical inbred strains and wild mice. **b** Diagram for constructing a mutant wild-like mouse model. **c** Screenshot of the passive tameness test from Additional file 3: Video S1 and Additional file 4: Video S2. **d** Passive tameness (accepting a touch of the hand of the operator) decreased significantly in female (*n* = 20 in each group, left panel) and male (*n* = 20 in each group, right panel) mutant mice. **e** Ratio of male individuals attacking (biting) the hand of the operator in tamed differs significantly from mutant mice. **f** The relative expression of total *Astn2* in tamed (*n* = 10 in each group) does not differ from mutant (*n* = 10 in each group) mice. **g** Diagram of the two *Astn2* isoforms (*a* and *b*) and the strategies of isoform-specific qPCR. Rectangles indicate *Astn2* exons, and red rectangles indicate the exon specially exist in the isoform *b*. **h** Ratio of *Astn2* isoform *a/b* in tamed (*n* = 10 in each group) and mutant (*n* = 10 in each group) mice. **i** Ratio of *Astn2* isoform *a/b* differs between classical inbred (*n* = 5 in each group) and wild (*n* = 5 in each group) mice. In panels **d**, **f**, **h**, and **i**, each circle indicates one individual of mouse, and error bars are SEM. * indicates *p* < 0.05

Based on our analysis on PSGs (see “Methods”), *Astn2* was used to construct a behavioral mouse model, although our results showed no differences in *Astn2* expression between wild mice and classical inbred strains (Additional file 1: Fig. S12). We then conducted an experiment to examine the relation of an SNP in the *Astn2* gene to the modification of mouse tameness. The SNP located at Chr4.66226438 (GRCm38.p2, intron of *Astn2*, rs27900929) exhibited a potentially strong selective signal (Fig. 4a). The frequency of the reference allele (T) of this SNP was only 4.17% in the wild mice, while the frequencies of the other two alleles (C and A) were 50.0% and 45.8%, respectively. In classical inbred mice, the frequency of the reference allele (C57BL/6J strain) was 79.3%, the frequency of allele A was 20.7%, and allele C completely disappeared.

To explore phenotypic effects associated with the SNP rs27900929, we used CRISPR-Cas9 strategy to mutate the allele T (Tamed mice) to C (Mutant mice) in C57BL/6J strain (Fig. 4b and Additional file 1: Fig. S13) and constructed a mutant mice strain. As expected, in the behavioral test of tameness (Fig. 4c, d and Additional file 1: Fig. S14, Table S21), the mutant mice showed a significant decrease (60–70%) of passive tameness as measured by accepting time (i.e., tolerance time) to the touch of a human hand [57]. In other words, the mutant mice often ran away or stretched their body to avoid the touch of human fingertips (Fig. 4c, d, Additional file 3: Video S1 and Additional file 4: Video S2). Approximately 30% of male mutant individuals attacked (bit) the hand of the tester, which was rarely observed in tamed mice (5%) (Fig. 4e and Additional file 5: Video S3). All these results indicated that the SNP located at Chr4. 66226438 (rs27900929) was associated with tameness in mice. Similar to the findings between classical inbred and wild mice mentioned above (Additional file 1: Fig. S12), the mutant mice exhibited no significant changes of *Astn2* expression in brain tissues as compared to tamed mice (Fig. 4f). This result suggests that there may be three ways in which the *Astn2* mutation affected tameness in mice. First, it may influence gene expression in special types of cells in the brain, so gene expression differences were hard to detect in total RNA from the brain tissue. Second, the mutation may influence gene expression in embryos or juveniles and change development, but not in adult mice. Third, the mutation may influence behavior via alternating gene structure and splicing, but not total gene expression.

### Intron mutation alters ratio of two *Astn2* isoforms

To detect the mechanisms of tameness alternation in mutant mice, we further explored the details of the mouse *Astn2* gene. *Astn2* has two alternative splicing mRNAs, isoform *a* and *b*. Isoform *a* is shorter than isoform *b* because of the exon 4 (156 bp, from 5′ end) deletion (Fig. 4g). We designed primers to detect *Astn2* isoform *a* and *b* specifically (Fig. 4g) and used qPCR to find whether the ratio of *Astn2* isoforms changed. The ratio of *Astn2* isoform *a/b* was significantly decreased by 20–30% in the mutant mice (Fig. 4h), consistent with the findings in wild mice and classical inbred strains (Fig. 4i). The accepting time showed an exponential increase with the ratio of *Astn2* isoform *a/b* (Additional file 1: Fig. S15), indicating that tameness was more associated with *Astn2* isoform *a*. By using AlphaFold2 [58] and other analyses, we found the structure and binding pockets of the two isoforms exhibited obvious differences (Additional file 1: Fig. S16), indicating there may be functional differences between the two splicing variants. As far as we know, this is the first evidence indicating that a single SNP triggers the functional modification (behavior) via alternative splicing in animal evolution. Alternative splicing is a vital force driving the evolution of animals [59–61] and here we discovered an SNP that may influence splicing approximately 100 kb downstream (Additional file 1: Fig. S13).

## Discussion

During the past decade, genome-wide strategies detected PSGs associated with behavior or nervous systems in many domesticated species [7–12, 62, 63]. The identified set of 339 PSGs in classic inbred mice of our study is closely associated with neurological functions, which is consistent with results published for model animal rat (*Rattus norvegicus*) living under almost identical conditions [13]. *Foxp2* and *Clock* were two of the PSGs associated with learning and circadian rhythms in rats, respectively [13]. In our study, *Foxp2* and *2310044G17Rik* (or “*Clock interacting protein, circadian*,: *Cipc*) were also identified in the 339 PSGs. The top gene functional category in the 339 PSGs, GABA-ergic synapses, also mirrored some of the genes in chicken domestication [64]. The discovery of the 339 PSGs in our study will benefit future studies in behavior or physiology of these genes in classic inbred mice.

Although many PSGs are found in domesticated animals, and some are closely associated with behavior traits [65, 66], few of their positive selected loci are strictly validated in mutant animal models. *Astn2* is highly expressed in the cerebellum and hippocampus [67]. It has been reported that *Astn2* attended neuronal migration [67] and surface protein trafficking [68]. *ASTN2* is also widely proven to be associated with a number of mental illnesses in humans [37–51] and has exhibited a relationship with hippocampus volume [69, 70]. A previous study showed that the double knockout of the exon 5 of *Astn2* and *Fz6* (*Frizzled6*) led to a 180° hair orientation reversal on the back of mice [71]; however, no behavioral phenotype of *Astn2* has yet been found in mutant or knockout mouse models. In this study, we built a point-mutation mouse model, and firstly detected that the SNP rs27900929 T > C was a positive selected locus, which increased passive tameness in the classical inbred mice (Fig. 4c, d, Additional file 3: Video S1 and Additional file 4: Video S2). Our results also support the view that the formation of tameness is dependent on a group of genes [63]. One SNP only triggered the modification of passive tameness (Fig. 4c, d, Additional file 3: Video S1 and Additional file 4: Video S2) but did not change other behavioral traits such as active tameness (Additional file 1: Fig. S14).

In previous studies, domestication has often been investigated by focusing on the variation of gene expression or the modification of amino acid sequence (non-synonymous SNP/mutation) [7, 65, 72]. In this study, we identified and detected shifts in expression variances occurring in *Kcnd2*, *Sebox*, and *Vwc2l* genes (Fig. 3d and Additional file 1: Fig S11). We found a different mechanism for rs27900929 in gene *Astn2* which affected the tameness and changed the ratio of different alternative splicing variants as well (Fig. 4). The product of the *Astn2* gene is a protein with two transmembrane regions, and the N- and C-terminals are both located outside the cell [73], leaving 150–200 amino acid residuals with intracellular location. The deleted exon 4 was located at the intracellular part and did not cause a frameshift, but prediction with AlphaFold2 [58] and other analyses indicated that the structure and binding pockets of the two isoforms showed obvious differences between each other (Additional file 1: Fig. S16). Thus, function of the *Astn2* isoform *a* and *b* proteins may be strongly modified by their structure, so as to alter individual behaviors. Alternative splicing is considered a major mechanism to enhance the diversity of transcriptome and proteome [59]. Growing evidence suggests that alternative splicing is a vital molecular mechanism of evolution [61] and development [74, 75] because it seems to contribute to novel traits [76, 77]. Our results further indicate that some SNPs may firstly reinforce one product of the alternative splicing under natural or artificial selection via SNPs or point mutations and finalize the functional changes via several steps under persistent selection pressure. It is still unknown as to how an SNP affects alternative splicing approximately 100 kb downstream (Additional file 1: Fig. S13). Although different alternative splicing was found to be closely associated with tameness modification in mice, the causal mechanism needs further investigation. Some other factors, such as trans-acting effects, may play a role in causing tameness modification.

Behavioral heterogeneity has been proven to exist among different mouse strains [78–80], and wild or domesticated genetic background altered the results of behavioral tests in the mutant mice model [81]. By using a large population of wild mice covering three subspecies, our results indicated that the classical inbred mice are distinctly clustered from all the wild mice and wild-derived inbred mice strains (Fig. 1), suggesting classical inbred mice were a mosaic of the three subspecies of wild mice and may have experienced very highly artificial selections. Genetic differences identified between classical inbred and wild mice are closely associated with the nervous system and behavior (Additional file 1: Fig. S7 and Additional file 2: Table S14) and may supply valuable implications for the studies of neuroethology. Furthermore, classical inbred mice are composed of several small clades (Fig. 1), so could provide useful genetic background information in behavioral or medical studies using classical inbred mice.

## Conclusion

By using resequencing genomic data of 36 wild mice, we identified 339 PSGs associated with domestication of the house mouse in laboratory conditions. GO analysis revealed that the PSGs are associated with membrane potential, transporter complex, and synapses. Approximately one third of these PSGs are highly expressed in the brain, and 125 genes exhibited abnormal phenotypes of behavior and in the nervous system. RNA-seq reveals that differentially expressed PSG genes were highly enriched in the hippocampus and frontal lobe. A mutant mouse model indicates that SNP rs27900929 (T > C) in gene *Astn2* regulates the tameness of mice through modifying the ratio of the two *Astn2* isoforms (*a/b*). Our results provide valuable cues for studying physiology and behaviors of animals using mouse models.

## Methods

### Ethics

Keeping and management of wild and laboratory mice included in this study followed the guidelines of Institute of Zoology and were approved by the Ethics Committee of the Institute of Zoology (IOZ20190048).

### Samples and sequencing

Totally 36 wild house mouse individuals were included in the experiment to explore genetic features of mice domestication, including 11 *M. m. domesticus*, 9 *M. m. musculus*, and 16 *M. m. castaneus* (Additional file 1: Table S1). Eleven samples of *M. m. domesticus* were captured in Germany (8 samples), Croatia (1 sample), Italy (1 sample), and UK (1 sample). The 8 samples from Germany were captured from the wild, and the other 3 were the offspring of wild mice after generation 3, 4, and 14 inbred mating. The 9 samples of *M. m. musculus* were obtained in Poland (1 sample), Czech Republic (1 sample), Russia (1 sample), and China (6 samples). The 16 samples of wild mice were captured in China. All samples of *M. m. musculus* and *M. m. castaneus* were acquired directly from the wild. Details of the wild mice are illustrated in Additional file 1: Fig. S1 and Table S1. The VCF files of 36 inbred laboratory mice were downloaded from the website of Sanger Institute [24].

Wild mice were captured using live traps (cages 23.5 cm × 11.5 cm × 11.5 cm) in China, transferred to a field laboratory, and then sacrificed after being anesthetized by isoflurane. The muscle samples for DNA extraction were snap frozen in liquid nitrogen and stored at −80 °C before DNA extraction. Genomic DNA was prepared using TIANamp Genomic DNA Kit (DP304, TIANGEN, Beijing China) following the manufacturer’s instructions. At least 10 μg genomic DNA of each sample was used to construct paired-end sequencing libraries, with the insert size of 300–400 base pairs according to Illumina DNA library preparation protocol. Then the libraries were sequenced using Illumina HiSeq 2000 and 4000.

### Variation calling and annotation

After quality filtering, the reads were mapped to the *Mus musculus* reference genome (GRCm38.p2) using BWA-MEM [25]. Single-nucleotide polymorphisms (SNPs) were individually detected by Genome Analysis Toolkit (GATK, ver 4.1.7) [82] HaplotypeCaller (gatk --java-options "-Xmx50G" HaplotypeCaller -R GRCm38.p2.fa -ERC GVCF -I $bam -O $gvcf --native-pair-hmm-threads 10). Individual GVCF files were combined using “CombineGVCFs,” and SNPs were genotyped and extracted by using “GenotypeGVCFs” and “SelectVariants.” The VCF file downloaded from Sanger Institute (36 inbred mouse strains) liftovered to GRCm38.p2 by using picard (Ver 2.20.5) LiftoverVcf (java -jar -Xmx50G -Djava.io.tmpdir=tmp picard.jar LiftoverVcf I=Sanger.snp.vcf O=Sanger_liftover.vcf CHAIN=GRCm38ToGRCm38.p2.over.chain REJECT=rejected_variants.vcf R=GRCm38.p2.fa). “VCF-merge” in VCFtools package [83] was used to merge the VCF file of the 36 wild mice and Sanger 36 inbred mouse strains. To provide empirically accurate base quality scores for each base in the read pairs, base quality recalibration was performed by GATK to reduce false positive rate. The criteria below were used to filter the raw SNPs: QD < 2.0; FS > 60.0; MQ < 40.0; HaplotypeScore > 13.0; ReadPosRankSum < –8.0; -cluster 3 -window 10. The statistics of the variants were calculated by in-house Python scripts. The variants are annotated with ANNOVAR (2019Oct24) [84].

### Phylogenetic relationship and population structure analysis

Totally 26,376,666 bi-allelic SNPs with miss <0.1 were enrolled in the phylogenetic relationship analysis. A phylogenetic tree was constructed by neighbor joining method (TreeBeST-1.9.2) [85] with 1000 bootstrap replicates among wild mice individuals, wild-derived inbred mice strains, and classical inbred mice strains. The result of tree construction was displayed using MEGA7 [86] and iTOL [87]. Population structure was conducted by the program ADMIXTURE (admixture_linux-1.23) [26] with the *K* values from 2 to 7 based on the cross-validation (CV) error (Additional file 1: Fig. S2). In order to reveal the relationships among the wild mice, wild-derived inbred mice, and classical inbred mice, a principal component analysis (PCA) was performed using GCTA64 [27] and plotted by in-house R scripts.

### Analysis of signatures of domesticated selection

The wild mice and wild-derived inbred mice were merged as a “wild group,” and the classical inbred mice were assigned as a “classical_inbred group.” Nucleotide diversity (*π*_wild_/*π*_classical_inbred_) and pairwise estimate of differentiation (Fst) were used to detect selected genes in domestication with the sliding windows of 40 kb size and 20 kb step. The VCF file was separated by a chromosome, and each chromosome was analyzed for XP-CLR score using XP-CLR (Ver 1.0), a dependent algorithm with XP-EHH, with parameters “-w1 0.005 200 2000 $chromosome -p0 0.95.” The average XP-CLR scores were calculated using 40-kb sliding window with a step size of 20 kb. The nucleotide diversities were calculated to acquire the ratio of *π*_wild_/*π*_classical_inbred_, the Fst values were calculated as described in Akey et al. [29], and XP-CLR values were estimated based on Chen et al. [30] in each window. The top 5% ranked windows in *π*_wild_/*π*_classical_inbred_, Fst, and XP-CLR scores were considered to be candidate selective regions. After annotated with ANNOVAR, the common genes selected by using the three approaches analysis were considered as positive selected genes (PSGs).

### Gene Ontology (GO) analysis

Gene Ontology (GO) analysis was performed using clusterProfiler (Ver 3.16.1) software package in R [88] with the database org.Mm.eg.db, and plotted by in-house R scripts. The functional categories with *p*-value less than 0.05 were considered statistically significant.

### Gene expression enriched analysis

To analyze expression levels of the 339 positively selected genes (PSGs), we downloaded the expression data (RPKM) in Mouse ENCODE transcriptome data (PRJNA66167) [31] from the NCBI website. The data includes a number of measurements in the tissues with similar functions, which might disturb the objectiveness of the calculation if the data was used directly. Hence, we merged these measurements and used their means in further analyses as follows: all the embryonic central neuro system tissues CNS E11.5, CNS E14, and CNS E18 were merged into “immature brain;” all the adult brain tissues cerebellum, cortex, and frontal lobe are merged into “brain;” all the embryonic liver tissues liver E14, liver E14.5, and liver E18 were merged into “immature liver;” duodenum, small intestine, large intestine, and colon are merged into “bowel”; spleen and thymus were merged into “immune system;” genital fat pad and subcutaneous fat pad were merged into “fat.” In total, 17 categories of tissues/organs were included in the analysis (Fig. 2f and Additional file 1: Fig. S8). For each gene, the average RPKM in the 17 categories of tissues/organs was calculated, and the genes with the RPKM at least 2-fold of the average RPKM in a tissues/organ were considered as “highly expressed genes.” We also set the data from CNS E11.5, CNS E14, CNS E18, cerebellum, cortex, and frontal lobe as immature brain/brain group, the data from other tissues as the other group, and used rank-sum test to measure the differences between the two groups.

### Phenotypes of mutant or knockout mouse model related to 339 PSGs

We compared our 339 PSGs with the phenotypes of behavior or nervous system in mutant or knockout mouse models downloaded from the web site of Mouse Genome Informatics (http://www.informatics.jax.org) [32]. The total genes with transgenic mouse models were counted based on the file “MGI_PhenotypicAllele.rpt.” The gene was defined as behavior/nervous system associated PSG if at least one of the relevant mutant or knockout mice showed the key words “behavior” and “nervous system” in descriptions of abnormal phenotypes. The mouse cell lines, simple reporter mice, and the mouse strains without clear gene annotation were excluded from our calculations.

### RNA extraction, library preparation, and RNA sequencing

The tissue RNA was extracted using TRIzol reagent (92008, Invitrogen, CA, USA) following the instructions. RNA integrity was assessed by the RNA Nano 6000 Assay Kit of the Bioanalyzer 2100 system (Agilent Technologies, CA, USA). NEBNext® Ultra™ RNA Library Prep Kit for Illumina® was used for the library preparation according to the instructions. Briefly, from total RNA, mRNA was purified using poly-T oligo-attached magnetic beads and then was fragmented using divalent cations. First-strand cDNA was synthesized using random hexamer primer and M-MuLV Reverse Transcriptase, and second-strand cDNA synthesis was subsequently performed using DNA Polymerase I. Adaptors were ligated to the double-strand cDNA. The cDNA fragments of 370–420 bps in length were purified with AMPure XP system (Beckman Coulter, Beverly, USA). PCR was performed to amplify the purified cDNA fragments with Phusion High-Fidelity DNA polymerase, Universal PCR primers, and Index (X) Primer. Finally, the PCR products were purified (AMPure XP system) again and library quality was assessed on the Agilent Bioanalyzer 2100 system. The library was then sequenced using Illumina Novaseq platform (Shanghai, China) and 150 bp paired-end reads were produced.

### Differential expression analysis

Differential expression analysis of two conditions/groups (at least two biological replicates per condition) was performed using the DESeq2 R package (1.20.0) [89]. The resulting *p* values were adjusted using the Benjamini and Hochberg’s approach for controlling the false discovery rate [90]. The gene with fragments per kilobase per million mapped reads (FPKM) > 1 is considered as expressed in the tissue. Genes with an adjusted *p*-value < 0.05 and FPKM > 1 were assigned as differentially expressed genes between wild mice and classical inbred mice. The proportion of differentially expressed genes of the 339 positively selected genes as well as of the total genes detected were calculated in hypothalamus, hippocampus, frontal lobe, heart, liver, and lung, separately.

### Gene selection for mouse model construction

The 339 PSGs were selected by three independent methods to minimize the influence of founder effect and genetic drift (Additional file 2: Table S13). They were used as the pool for candidate gene selection. We excluded genes that had been proven to have nervous system or behavioral phenotypes in genetic mouse models from the 339 PSGs (Additional file 2: Table S17), focusing on genes with unknown behavioral phenotypes. We did not refer to the results of RNA-seq and real-time PCR in selecting candidate genes. In fact, a high ratio of expression significantly changed PSGs was an effective way to demonstrate the artificial selection that had affected these PSGs. However, for one single or several genes used to construct mouse models, RNA expression was weakly associated with both genotypes and phenotypes.

We did two analyses in selecting the genes and SNP sites. In the first analysis, we did not have samples of wild subspecies *M. m. castaneus* but performed an analysis to select genes by using wild mice of two subspecies *M. m. domesticus* and *M. m. musculus*. We found eight genes that exhibited a close relationship to mental illness and behavior, including *Prkcq*, *Astn2*, *Gm20388*, *Pcdh15*, *Eea1*, *Nav3*, *Nrxn3*, and *Iglc2*. We selected the sites for gene editing and behavioral tests using the simple rule that reference allele homozygosity does not exist in any wild mice (*n*=21), but exists in all the classical inbred mice (*n*=28). A total of 32 sites were found, including 4 sites in *Astn2*, 1 site in *Gm20388*, 1 site in *Eea1*, 3 sites in *Nav3*, and 23 sites in *Nrxn3* (Additional file 2: Table S22). We constructed four mouse models (Additional file 1: Table S23) and found only *Astn2* showed a significant difference in behavioral performance between wild and mutant types at the SNP rs27900929.

Later on, we succeeded in capturing mice belonging to the wild subspecies *M. m. castaneus*. Then, we re-ran the selection process by including three wild subspecies of *M. m. domesticus*, *M. m. musculus* and *M. m. castaneus*. We found the genome sequencing quality of one wild *M. m. musculus* was not high and so it was excluded in the second analysis. We found only *Astn2*, *Pcdh15*, and *Nrxn3* remained in the top selected genes. However, we were not able to select the SNP of *Astn2* located at Chr4: 66226438 using the original rule. Of this SNP, the frequency of reference allele T was 0.0417 in wild mice, and 0.793 in inbred mice. After further investigation following the first part of the rule, that is, no reference allele homozygous exists in wild mice, we found the SNP located at Chr4: 66226438 was included in the selected 69 SNPs of *Astn*2 (Additional file 2: Table S24). Thus, the original rule (reference allele homozygous does not exist in any wild mice, but exists in all the classical inbred mice) would be extreme in selecting the potential selected SNPs under domestication. Here, we developed a modified criterion to select the potentially interesting SNPs: the frequency of the reference allele in the wild mice should be less than 20% of the frequency in classical inbred mice. The criterion is similar to the original one in principle, but with a much more relaxed condition than the original rule. Using the new criterion, we obtained 196 SNPs (Additional file 2: Table S25) from in total 11,017 SNPs located at *Astn2*. Among the 196 SNPs, 7 SNPs were tri-allelic and 189 SNPs were bi-allelic in wild mice; 1 SNPs was tri-allelic, 161 SNPs were bi-allelic, and 34 SNPs were with single allele in classical inbred mice (Additional file 1: Fig. S17). The SNP of Chr4: 66226438 was located in tri-allele in wild mice and bi-allele in classical inbred mice.

The function and their relation to the behavior of *Pcdh15* and *Nrxn3* have been well studied and were not used in constructing the mouse model. Limited information is known as to the function and relation to the behavior of *Astn2*, *Prkcq*, and *Eea1*, which were used for constructing the mouse model. Only *Astn2* was found to show behavioral differences between wild and mutant mice. We did not complete behavioral assessment of some mouse models due to inadequate sample size (For details, please see Additional file 1: Table S23).

### Construction of the mouse model

The point-mutation mouse model (*Astn2* 66226438 T > C) was constructed via CRISPR-Cas9 strategy. Briefly, Cas9 mRNA, gRNA, and donor DNA were co-injected in fertilized eggs of the C57BL/6J strain. The injected eggs were cultured overnight in kSOM and transferred back into pseudopregnant female mice to acquire F_0_ mice. The tamed-type mice (T/T) and wild-type (C/C) mice were identified via PCR and the following sequencing (Additional file 1: Fig. S13). The reaction of the PCR for mice identification was at 94 °C for 5 min, followed by 35 cycles of 94 °C for 30 s, 56 °C for 30 s, and 72 °C for 1 min. The mouse model generation was assigned to Shanghai Model Organisms. The primers of mouse genotype identification are shown in Additional file 1: Table S26.

### Identification of tameness

The tameness of mice was measured via the method described in Nagayama et al. [57] with some modifications. Tameness has two behavioral components: active tameness and passive tameness. Active tameness is referred to the animal actively approaching/contacting human hands, and passive tameness means the tolerance of the animal to touching by human hands [57]. We used a hand to test the reaction of mice in a gray plastic box of 40 × 40 × 40 cm. Mice were not touched by hand 24 h before the test. Before and between tests, touching the mice was prohibited; instead, long tweezers were used. The tips of the long tweezers were covered with silicon tubes to avoid hurting the mice. The first tameness test was conducted to measure active tameness. When the test started, the operator placed one mouse in the middle of the box with a pair of long tweezers and put his/her left hand on the bottom of the box with palm up, moving towards the mouse until a distance of about 10 cm between the fingertips and the mouse was achieved. The operator kept a distance of 10 cm from the mouse when the mouse moved away from the hand. The time of active contacting of the mice to the hand was recorded as the measure of active tameness in the mice. The active tameness test lasted for 1 min.

The second tameness test was conducted to measure passive tameness of mice. This test started just after the active tameness test. The mouse was placed in the middle of the box with tweezers and the operator put his/her left hand on the bottom of the box with palm up, moving towards the mouse until the fingertips gently touched the mouse. The operator kept the hand on the mouse until the end of the test. The test lasted for 1 min. The time of the passive acceptance (i.e., accepting time) of the mouse to the hand was recorded to measure the passive tameness of the mice. The “acceptance” to the touch of a hand was defined as a period time more than 0.5 s, during which time the mouse did not exhibit behavior of moving away from the hand (such as running away or stretching the body). The active touch of the mouse to the hand was also considered as “acceptance” to the touch of the hand. The frequency of attacking the hand was also recorded. All the measurements were recorded by a video recorder and analyzed via tanaMove software (V0.01). Significant differences of active or passive tameness were detected by two-tailed Student’s *t* test.

### Animal sacrificing and tissue storage

The mice were anaesthetized by isoflurane first, and blood was collected before they were sacrificed. The hypothalamus, hippocampus, frontal lobe, heart, liver, and lung tissues were collected and moved into tubes of RNase-free. The tubes were snap frozen in liquid nitrogen and kept in liquid nitrogen until use.

### Reverse-transcription and real-time quantitative polymerase chain reaction (qPCR)

The RNA was extracted from the frozen tissues stored in liquid nitrogen using TRIzol reagent (92008, Invitrogen, CA, USA) following the instructions. The extracted RNAs were dissolved in RNase-free distilled water (W4502, Sigma-Aldrich, MO, US), and totally 2 μg RNA was used for the following reverse-transcription. The reverse-transcription was performed using RevertAid First Strand cDNA Synthesis Kit (K1622, Thermo Scientific, Shanghai, China) according to the instructions. The cDNA samples were stored at −80 °C until use.

The qPCR was performed using TB Green Premix Ex Taq II (Tli RNase H Plus) (RR820, Takara, Beijing, China) on a Thermo Scientific PikoReal Real-Time PCR System (Thermo Scientific, Shanghai, China) in a total volume of 10 μl. *Gapdh* was used as the reference housekeeping gene. The reaction of samples was set at 95 °C for 7 min, followed by 40 cycles of 95 °C for 5 s, and 60 °C for 30 s. The method of 2^−△△Ct^ was used to calculate the fold change of gene expression. The index of *Astn2* isoform *a/b* was calculated similarly as the 2^−△△Ct^ method. The *Astn2* isoform *a* was used as the measurement gene, and the *Astn2* isoform *b* was used as the reference gene. For each gene of each sample, the experiment was performed in triplicate. The primers for qPCR are shown in Additional file 1: Table S26. Significant differences of gene expression or gene ratio are detected by using two-tailed Student’s *t* test.

### Protein structure prediction

Models of *Astn2* isoform *a* were generated using a local copy of AlphaFold2 (Ver 2.1.1) [58] with full_dbs preset, open-source code available at https://github.com/deepmind/alphafold. Runs were performed on a CentOS 7.8.2003 workstation with 320 GB RAM, 80 CPUs and a NVIDIA Tesla V100 SXM2 32GB GPU card. The full-length structure of *Astn2* isoform *b* were downloaded from DeepMind AlphaFold2 database hosted at EBI (https://alphafold.ebi.ac.uk/files/AF-Q80Z10-F1-model_v2.pdb.). Online tool POCASA (http://g6altair.sci.hokudai.ac.jp/g6/service/pocasa/) [91] was used to predict the binding pockets of two proteins, and default parameters were used for analysis. Protein structure visualizations were created in PyMOL Open-Source build v.2.6.0 (https://github.com/schrodinger/pymol-open-source) [92].

## Supplementary Information

Additional file 1: Figure S1. Mouse samples in this study. a. Geographical sites of the 36 wild mice. b. The definition and relation of wild, wild-derived inbred and classic inbred mice in this study. Figure S2. The CV error variation for different K values of ADMIXTURE analysis on genomes of mice. Figure S3. PCA analysis on mouse genomes. a. The three-dimensional diagram of PCA based on the top three principal components (PC1-PC3). b. The percentage of eigenvalue in the top 10 principal components. Figure S4. Top 20 GO categories of the genes located in low nucleotide diversity regions (top 5%) in classical inbred mice as compared with those in wild mice and wild-derived inbred mice. Figure S5. Top 20 GO categories of the genes located in top 5% Fst regions in classical inbred mice as compared with those of wild mice and wild-derived inbred mice. Figure S6. Top 20 GO categories of the genes located in top 5% XP-CLR regions in classical inbred mice as compared with those of wild mice and wild-derived inbred mice. Figure S7. Top 20 GO categories of the 339 common positive selected genes in classical inbred mice as compared with those of wild mice and wild-derived inbred mice. Figure S8. The ratio of highly expressed genes in different organs and tissues in mice. The ratio of highly expressed genes in the immature brain, brain, liver, heart, and lung are illustrated in Fig. 2f. Figure S9. The ratio of the genes with abnormal behavioral phenotypes in mouse models. Figure S10. The heatmap of the 56 common differently expressed genes (merged from hippocampus and frontal lobe) in the frontal lobe of mice. Figure S11. The differences in relative expression of *Vwc2l* between classical inbred and wild mice. Each circle indicates one individual mouse, and error bars are standard error of mean (SEM). * indicates *p* < 0.05. Figure S12. The differences in relative expression of *Astn2* between classical inbred and wild mice. Each circle indicates one individual mouse, and error bars are SEM. Figure S13. The mutant mice model of rs27900929 in *Astn2* gene. a. The position of rs27900929 in the *Astn2* gene. The rectangles indicate exons of *Astn2*, and the red rectangles indicate the exon specially exist in the isoform *b*. b. The identification of *Astn2* mutant mice. The bands for sequencing is 1085 bp. Red arrow heads indicate the mutant position. Figure S14. The differences in active tameness (actively contacting the hand of operators) between tamed and mutant mice. Each circle indicates one individual mouse, and error bars are SEM. Figure S15. The exponential relationship between accepting time and ratio of *Astn2* isoform *a/b*. Each circle indicates one individual mouse. Passive tameness was the tolerance of the animal to touch from a human hand, as measured by accepting time. Figure S16. The binding pockets of the proteins of the *Astn2* isoform *a* and *b*. The red color indicates the different area (Exon 4), and the yellow color indicates the large pockets. Isoform *a* lacks an alpha helix, and there is a binding pocket nearby the alpha helix of isoform *b*. Figure S17. The frequency and their relationship of tri-, bi- and single allele of SNPs of *Astn2* in wild and classical inbred mice by using the criterion that the frequency of the reference allele in the wild mice is less than 20% of that in classical inbred mice. Table S1. The characters of the 36 wild mouse samples. Table S2. The characters of the 36 inbred mouse strains downloaded from Sanger Institute. Table S3. The sequencing characters of the 36 wild mouse samples. Table S4. Number of raw SNPs and their distributions in wild and classical inbred mice. Table S5. Number of raw SNPs and their distributions in wild-derived inbred mice originating from *M. musculus*. Table S6. Wild-derived inbred mice and their wild relatives. Table S21. Details of the tameness test in tamed and mutant mice. Table S23. Constructed mouse models for tameness test. Table S26. Primers used in this study.

Additional file 2: Table S7. Genes located in low nucleotide diversity regions (top 5%) in classical inbred mice strains as compared to wild and wild-derived inbred mice. Table S8. Functional categories of the genes (*p* < 0.05) located at low nucleotide diversity regions (top 5%) in classical inbred mice strains as compared to wild and wild-derived inbred mice. Table S9. Genes located in top 5% Fst regions in classical inbred mice strains as compared to wild and wild-derived inbred mice. Table S10. Functional categories of the genes (*p* < 0.05) located in top 5% Fst regions in classical inbred mice strains compared to wild and wild-derived inbred mice. Table S11. Genes with top 5% XP-CLR score in classical inbred mice strains as compared to wild and wild-derived inbred mice. Table S12. Functional categories of the genes (*p* < 0.05) with top 5% XP-CLR score in classical inbred mice strains as compared to wild and wild-derived inbred mice. Table S13. Common 339 positively selected genes (PSGs) in classical inbred mice strains as compared to wild and wild-derived inbred mice. Table S14. Functional categories of the common 339 positively selected genes in classical inbred mice strains as compared to wild and wild-derived inbred mice. Table S15. Common 355 positively selected genes between classical inbred mice strains and wild mice after excluding wild-derived inbred mice. Table S16. Genes significantly higher expressed in immature brain/brain than in other tissues. Table S17. Abnormal phenotypes of the 245 genes from 339 PSGs reported in mouse models. Table S18. Differently expressed genes of the 339 PSGs in the hippocampus between classical inbred and wild mice. Table S19. Differently expressed genes of the 339 PSGs in the frontal lobe between classical inbred and wild mice. Table S20. Differently expressed genes of the 339 PSGs in the hypothalamus between classical inbred and wild mice. Table S22. Selected sites with reference allele homozygous not existing in any wild mice, but existing in all the classical inbred mice. Table S24. SNPs located in *Astn2* gene with no homozygous of reference allele in wild mice. Table S25. SNPs located in *Astn2* gene with high selective potential.

Additional file 3: Video S1. The video of a tamed (T/T) mouse.

Additional file 4: Video S2. The video of a mutant (C/C) mouse.

Additional file 5: Video S3. The video of a mutant (C/C) mouse biting the operator.

Additional file 6. Review history.

## Data Availability

The raw sequence data reported in this manuscript (including 36 wild mice whole genome resequencing; 30 samples of wild mice for RNA-Seq; 30 samples of tamed mice for RNA-Seq) has been deposited in the Genome Sequence Archive in National Genomics Data Center, China National Center for Bioinformation / Beijing Institute of Genomics, Chinese Academy of Sciences (GSA: CRA008086) that are publicly accessible at https://ngdc.cncb.ac.cn/gsa [93]. The genomic resequencing data of the 36 inbred laboratory mice (VCF files) was downloaded from the website of Sanger Institute [24].
