# Supplementary material for: Whole-genome sequencing reveals the genetic mechanisms of domestication in classical inbred mice
Source: Genome Biol. 2022 Sep 26;23:203. doi: 10.1186/s13059-022-02772-1 (PMC9511766; doi:10.1186/s13059-022-02772-1)
Supplement: Supplementary file 1 — Additional file 1: Figure S1. Mouse samples in this study. a. Geographical sites of the 36 wild mice. b. The definition and relation of wild, wild-derived inbred and classic inbred mice in this study. Figure S2. The CV error variation for different K values of ADMIXTURE analysis on genomes of mice. Figure S3. PCA analysis on mouse genomes. a. The three-dimensional diagram of PCA based on the top three principal components (PC1-PC3). b. The percentage of eigenvalue in the top 10 principal components. Figure S4. Top 20 GO categories of the genes located in low nucleotide diversity regions (top 5%) in classical inbred mice as compared with those in wild mice and wild-derived inbred mice. Figure S5. Top 20 GO categories of the genes located in top 5% Fst regions in classical inbred mice as compared with those of wild mice and wild-derived inbred mice. Figure S6. Top 20 GO categories of the genes located in top 5% XP-CLR regions in classical inbred mice as compared with those of wild mice and wild-derived inbred mice. Figure S7. Top 20 GO categories of the 339 common positive selected genes in classical inbred mice as compared with those of wild mice and wild-derived inbred mice. Figure S8. The ratio of highly expressed genes in different organs and tissues in mice. The ratio of highly expressed genes in the immature brain, brain, liver, heart, and lung are illustrated in Fig. 2f. Figure S9. The ratio of the genes with abnormal behavioral phenotypes in mouse models. Figure S10. The heatmap of the 56 common differently expressed genes (merged from hippocampus and frontal lobe) in the frontal lobe of mice. Figure S11. The differences in relative expression of Vwc2l between classical inbred and wild mice. Each circle indicates one individual mouse, and error bars are standard error of mean (SEM). * indicates p < 0.05. Figure S12. The differences in relative expression of Astn2 between classical inbred and wild mice. Each circle indicates one individual mouse, and error bars [file 13059_2022_2772_MOESM1_ESM.docx]

**Whole-genome sequencing reveals the genetic mechanisms of domestication in classical inbred mice**

**Supplementary Information**

**Additional file 1:** The file “Additional file 1.docx” includes Fig S1–S17, Table S1–S6, Table S21, Table S23, and Table S26.

**Fig. S1.** Mouse samples in this study. a. Geographical sites of the 36 wild mice. b. The definition and relation of wild, wild-derived inbred and classic inbred mice in this study.

**Fig. S2.** The CV error variation for different K values of ADMIXTURE analysis on genomes of mice.

**Fig. S3.** PCA analysis on mouse genomes. a. The three-dimensional diagram of PCA based on the top three principal components (PC1-PC3). b. The percentage of eigenvalue in the top 10 principal components.

**Fig. S4.** Top 20 GO categories of the genes located in low nucleotide diversity regions (top 5%) in classical inbred mice as compared with those in wild mice and wild-derived inbred mice.

**Fig. S5.** Top 20 GO categories of the genes located in top 5% Fst regions in classical inbred mice as compared with those of wild mice and wild-derived inbred mice.

**Fig. S6.** Top 20 GO categories of the genes located in top 5% XP-CLR regions in classical inbred mice as compared with those of wild mice and wild-derived inbred mice.

**Fig. S7.** Top 20 GO categories of the 339 common positive selected genes in classical inbred mice as compared with those of wild mice and wild-derived inbred mice.

**Fig. S8.** The ratio of highly expressed genes in different organs and tissues in mice. The ratio of highly expressed genes in the immature brain, brain, liver, heart, and lung are illustrated in Fig. 2f.

**Fig. S9.** The ratio of the genes with abnormal behavioral phenotypes in mouse models.

**Fig. S10.** The heatmap of the 56 common differently expressed genes (merged from hippocampus and frontal lobe) in the frontal lobe of mice.

**Fig. S11.** The differences in relative expression of *Vwc2l* between classical inbred and wild mice. Each circle indicates one individual mouse, and error bars are standard error of mean (SEM). * indicates *p* < 0.05.

**Fig. S12.** The differences in relative expression of *Astn2* between classical inbred and wild mice. Each circle indicates one individual mouse, and error bars are SEM.

**Fig. S13.** The mutant mice model of rs27900929 in *Astn2* gene. a. The position of rs27900929 in the *Astn2* gene. The rectangles indicate exons of *Astn2*, and the red rectangles indicate the exon specially exist in the isoform *b*. b. The identification of *Astn2* mutant mice. The bands for sequencing is 1085 bp. Red arrow heads indicate the mutant position.

**Fig. S14.** The differences in active tameness (actively contacting the hand of operators) between tamed and mutant mice. Each circle indicates one individual mouse, and error bars are SEM.

**Fig. S15.** The exponential relationship between accepting time and ratio of *Astn2* isoform *a/b*. Each circle indicates one individual mouse. Passive tameness was the tolerance of the animal to touch from a human hand, as measured by accepting time.

**Fig. S16.** The binding pockets of the proteins of the *Astn2* isoform *a* and *b*. The red color indicates the different area (Exon 4), and the yellow color indicates the large pockets. Isoform *a* lacks an alpha helix, and there is a binding pocket nearby the alpha helix of isoform *b*.

**Fig. S17.** The frequency and their relationship of tri-, bi- and single allele of SNPs of *Astn2* in wild and classical inbred mice by using the criterion that the frequency of the reference allele in the wild mice is less than 20% of that in classical inbred mice.

**Table S1.** The characters of the 36 wild mouse samples.

**Table S2.** The characters of the 36 inbred mouse strains downloaded from Sanger Institute.

**Table S3.** The sequencing characters of the 36 wild mouse samples.

**Table S4.** Number of raw SNPs and their distributions in wild and classical inbred mice.

**Table S5.** Number of raw SNPs and their distributions in wild-derived inbred mice originating from *M. musculus*.

**Table S6.** Wild-derived inbred mice and their wild relatives.

**Table S21.** Details of the tameness test in tamed and mutant mice.

**Table S23.** Constructed mouse models for tameness test.

**Table S26.** Primers used in this study.

**Additional file 2:** The file “Additional file 2.xlsx” includes Table S7–S20, Table S22, Table S24, and Table S25, large tables.

**Table S7.** Genes located in low nucleotide diversity regions (top 5%) in classical inbred mice strains as compared to wild and wild-derived inbred mice.

**Table S8.** Functional categories of the genes (*p* < 0.05) located at low nucleotide diversity regions (top 5%) in classical inbred mice strains as compared to wild and wild-derived inbred mice.

**Table S9.** Genes located in top 5% Fst regions in classical inbred mice strains as compared to wild and wild-derived inbred mice.

**Table S10.** Functional categories of the genes (*p* < 0.05) located in top 5% Fst regions in classical inbred mice strains compared to wild and wild-derived inbred mice.

**Table S11.** Genes with top 5% XP-CLR score in classical inbred mice strains as compared to wild and wild-derived inbred mice.

**Table S12.** Functional categories of the genes (*p* < 0.05) with top 5% XP-CLR score in classical inbred mice strains as compared to wild and wild-derived inbred mice.

**Table S13.** Common 339 positively selected genes (PSGs) in classical inbred mice strains as compared to wild and wild-derived inbred mice.

**Table S14.** Functional categories of the common 339 positively selected genes in classical inbred mice strains as compared to wild and wild-derived inbred mice.

**Table S15.** Common 355 positively selected genes between classical inbred mice strains and wild mice after excluding wild-derived inbred mice.

**Table S16.** Genes significantly higher expressed in immature brain/brain than in other tissues.

**Table S17.** Abnormal phenotypes of the 245 genes from 339 PSGs reported in mouse models.

**Table S18.** Differently expressed genes of the 339 PSGs in the hippocampus between classical inbred and wild mice.

**Table S19.** Differently expressed genes of the 339 PSGs in the frontal lobe between classical inbred and wild mice.

**Table S20.** Differently expressed genes of the 339 PSGs in the hypothalamus between classical inbred and wild mice.

**Table S22.** Selected sites with reference allele homozygous not existing in any wild mice, but existing in all the classical inbred mice.

**Table S24.** SNPs located in *Astn2* gene with no homozygous of reference allele in wild mice.

**Table S25.** SNPs located in *Astn2* gene with high selective potential.

**Additional file 3:** The file “Additional file 3.mp4” is Video S1, the video of a tamed (T/T) mouse.

**Additional file 4:** The file “Additional file 4.mp4” is Video S2, the video of a mutant (C/C) mouse.

**Additional file 5:** The file “Additional file 5.mp4” is Video S3, the video of a mutant (C/C) mouse biting the operator.

**
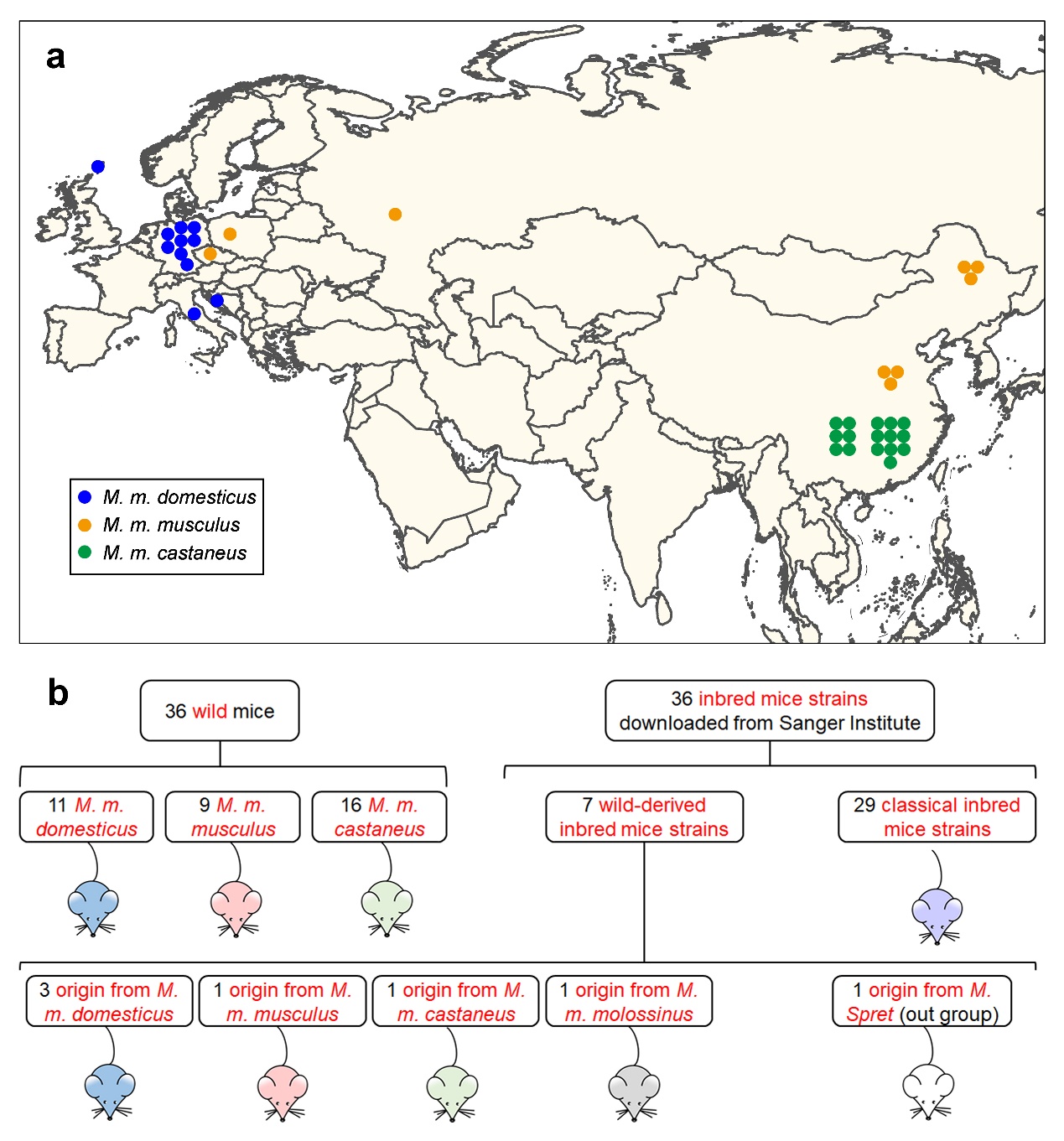
**

**Fig. S1.** Mouse samples in this study. a. Geographical sites of the 36 wild mice. b. The definition and relation of wild, wild-derived inbred and classic inbred mice in this study.

**
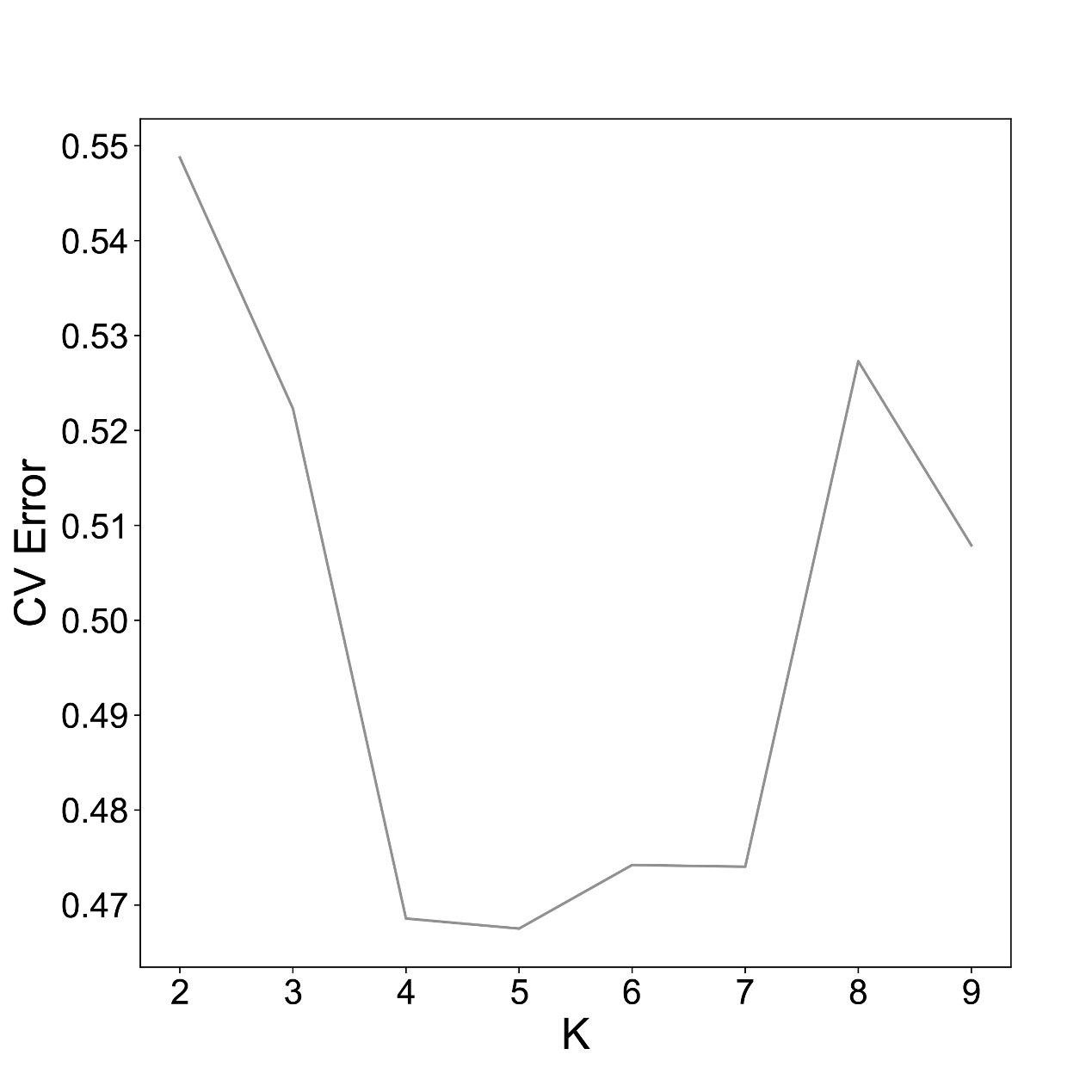
**

**Fig. S2.** The CV error variation for different K values of ADMIXTURE analysis on genomes of mice.

**
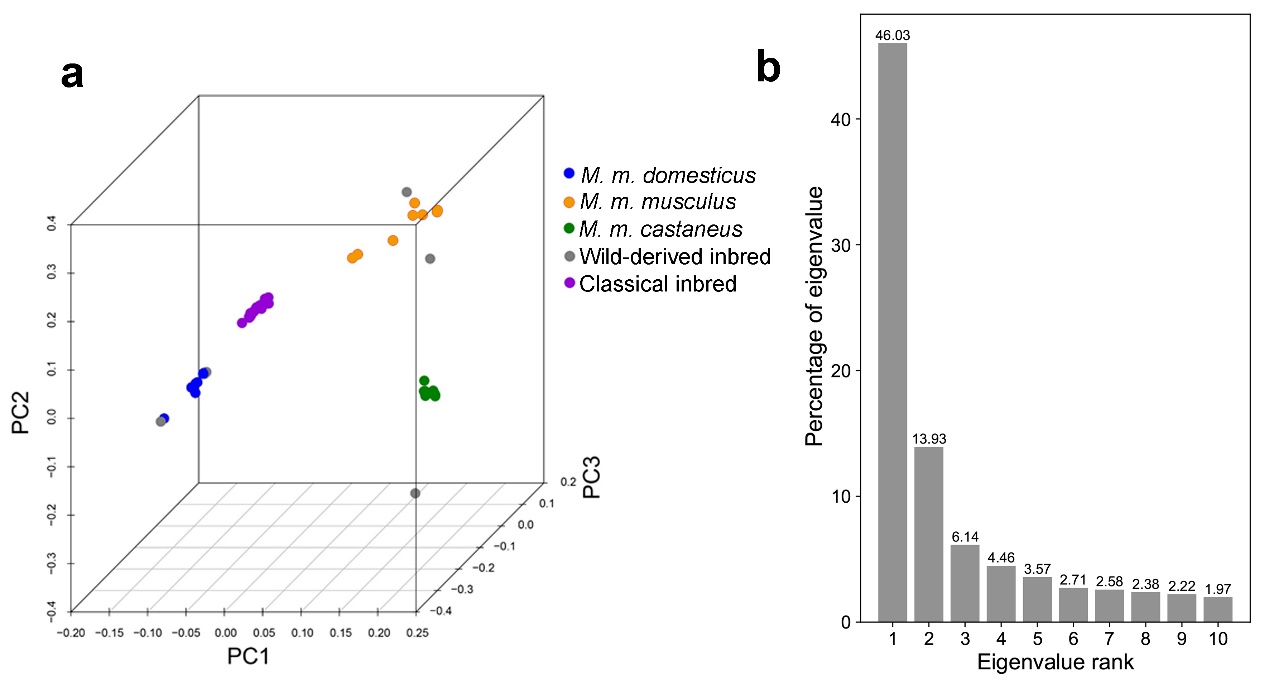
**

**Fig. S3.** PCA analysis on mouse genomes. a. The three-dimensional diagram of PCA based on the top three principal components (PC1-PC3). b. The percentage of eigenvalue in the top 10 principal components.

**
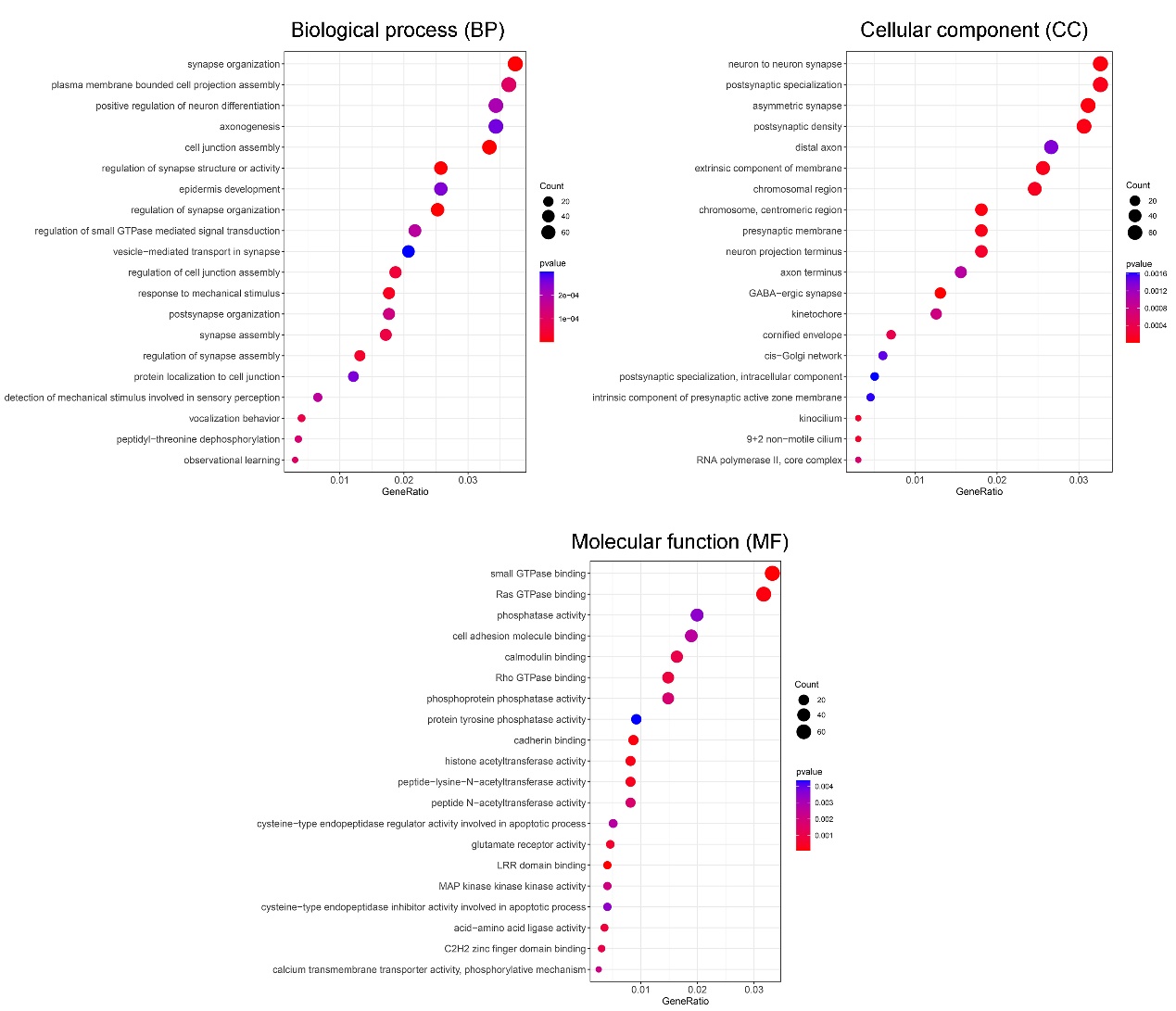
**

**Fig. S4.** Top 20 GO categories of the genes located in low nucleotide diversity regions (top 5%) in classical inbred mice as compared with those in wild mice and wild-derived inbred mice.

**
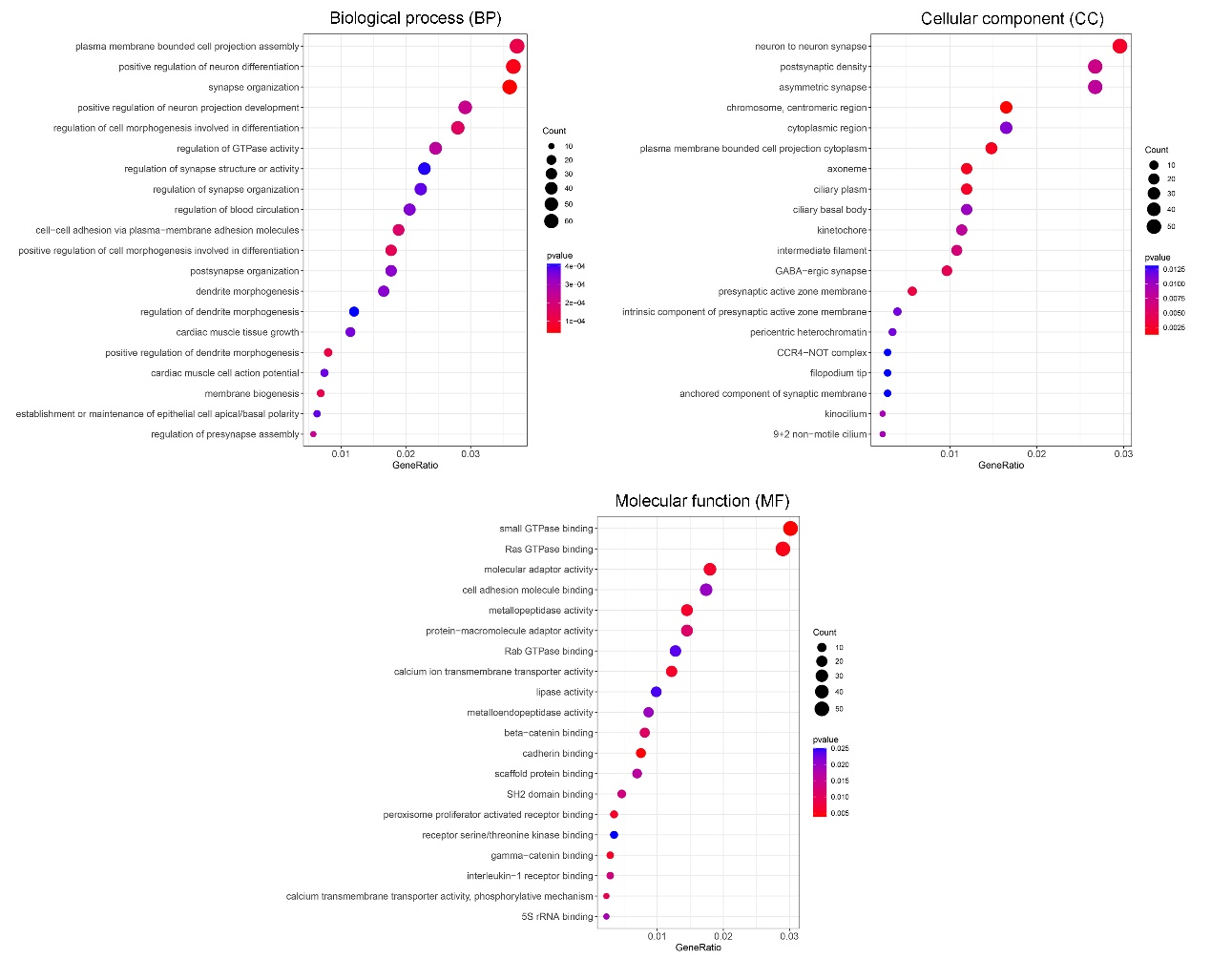
**

**Fig. S5.** Top 20 GO categories of the genes located in top 5% Fst regions in classical inbred mice as compared with those of wild mice and wild-derived inbred mice.

**
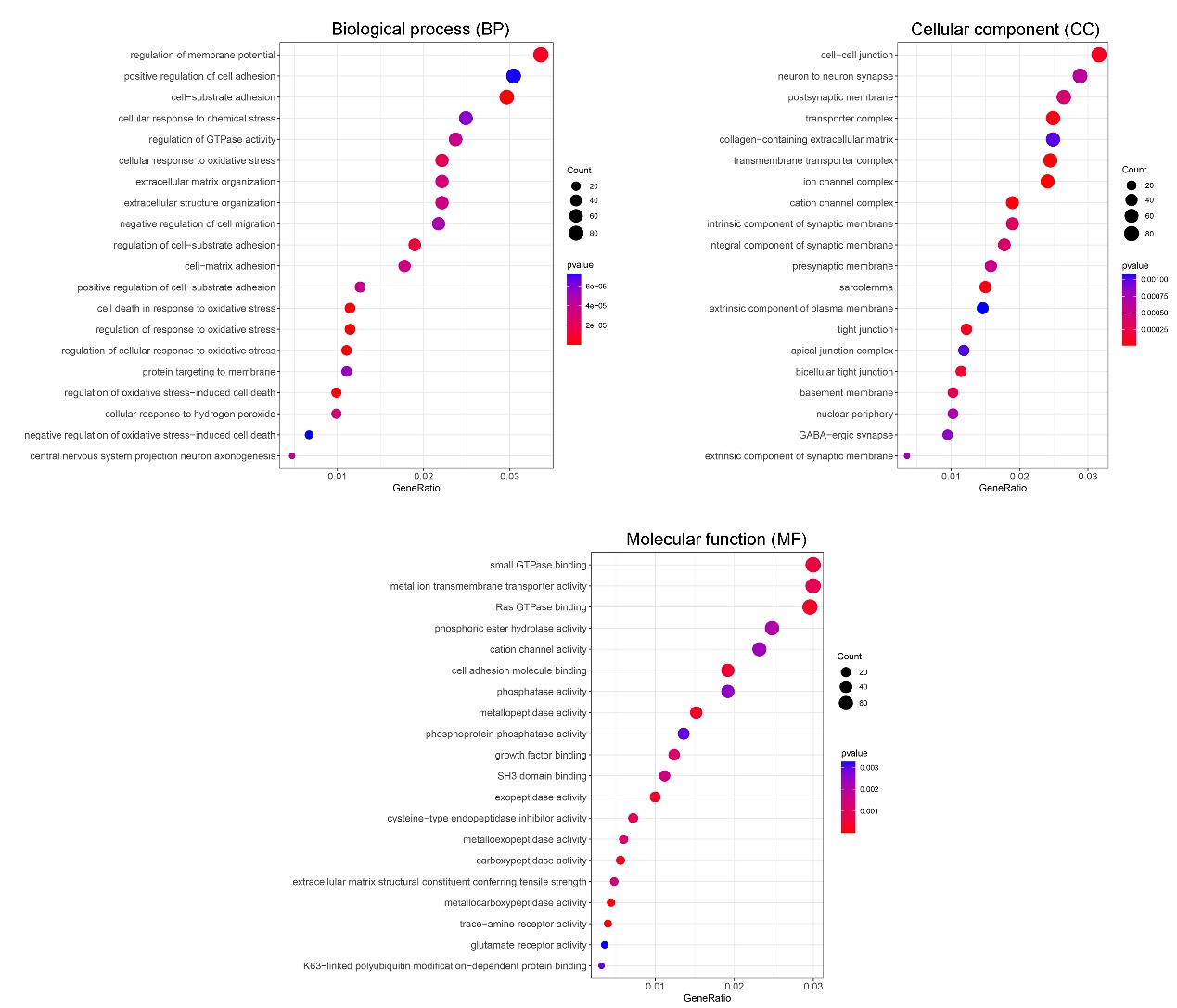
**

**Fig. S6.** Top 20 GO categories of the genes located in top 5% XP-CLR regions in classical inbred mice as compared with those of wild mice and wild-derived inbred mice.

**
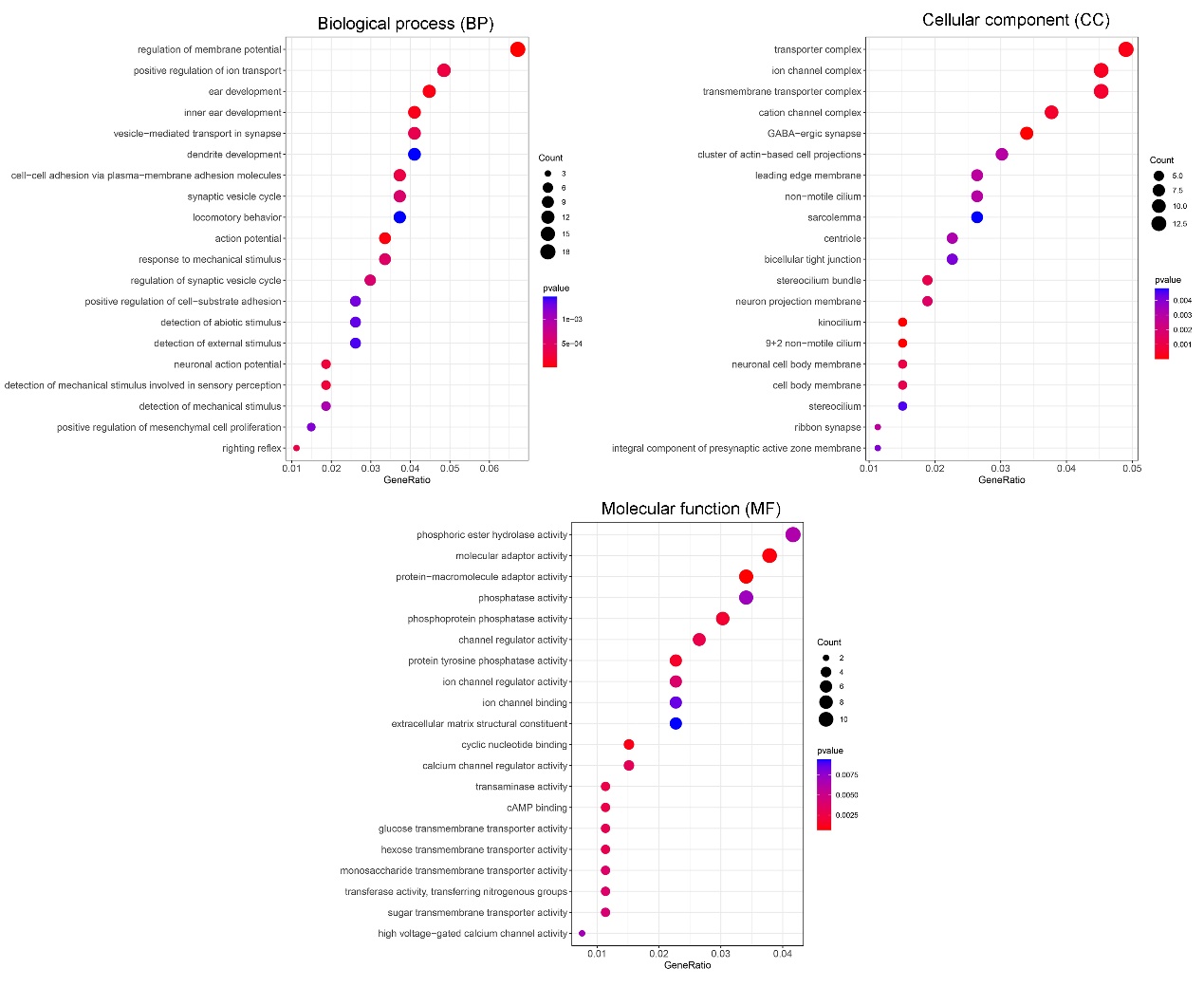
**

**Fig. S7.** Top 20 GO categories of the 339 common positive selected genes in classical inbred mice as compared with those of wild mice and wild-derived inbred mice.

**
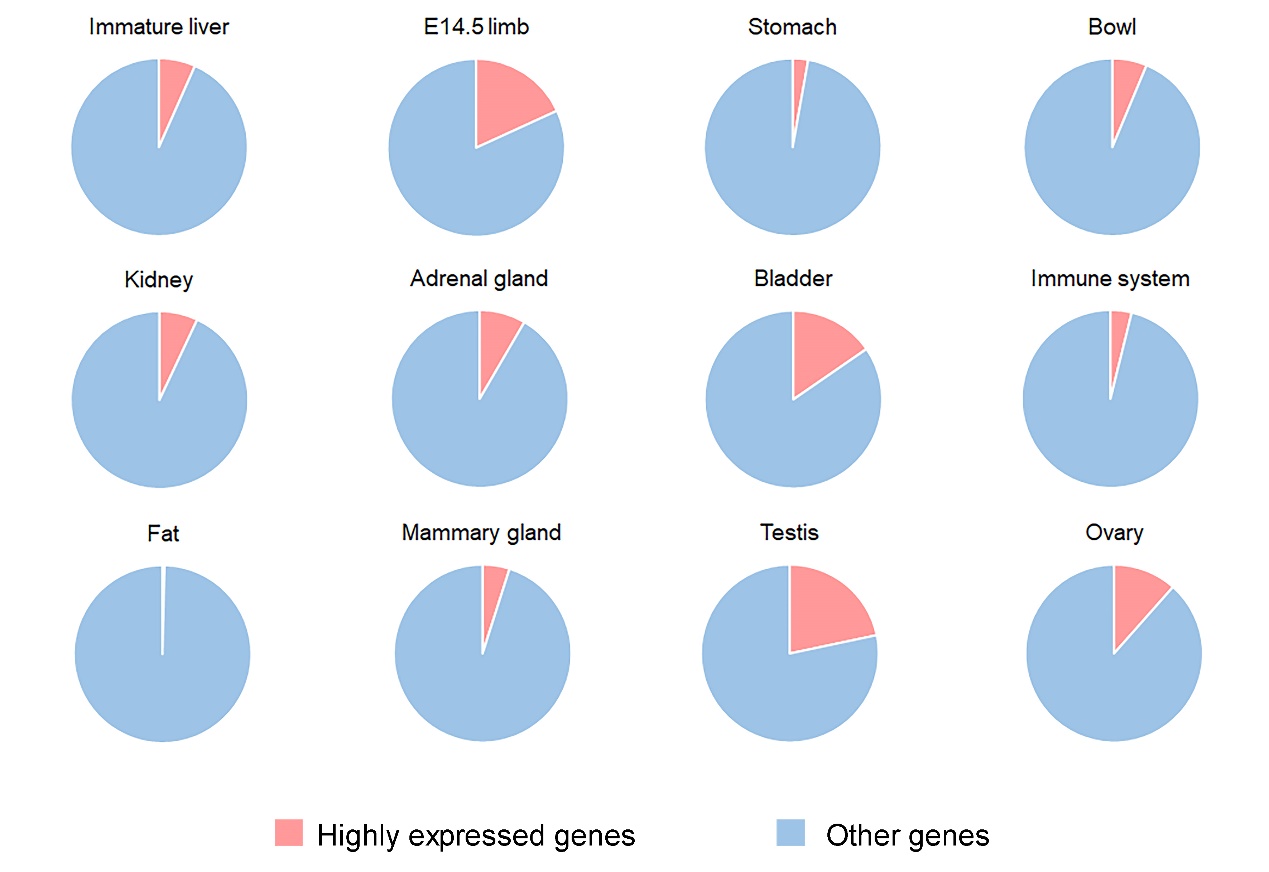
**

**Fig. S8.** The ratio of highly expressed genes in different organs and tissues in mice. The ratio of highly expressed genes in the immature brain, brain, liver, heart, and lung are illustrated in Fig. 2f.

**
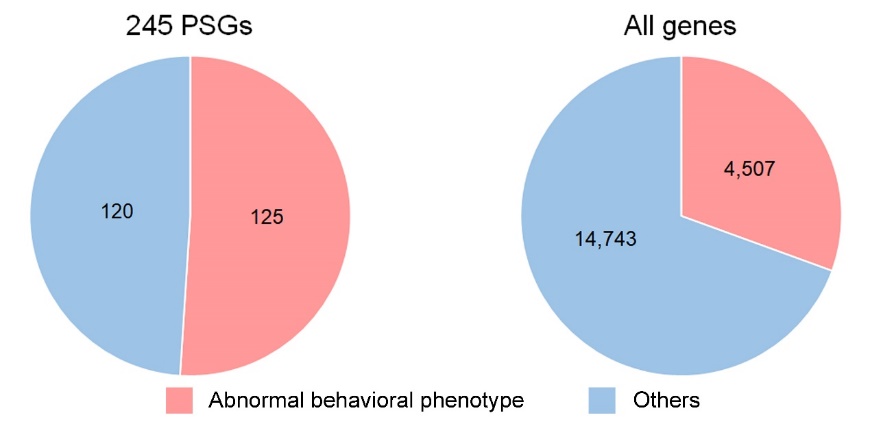
**

**Fig. S9.** The ratio of the genes with abnormal behavioral phenotypes in mouse models.

**
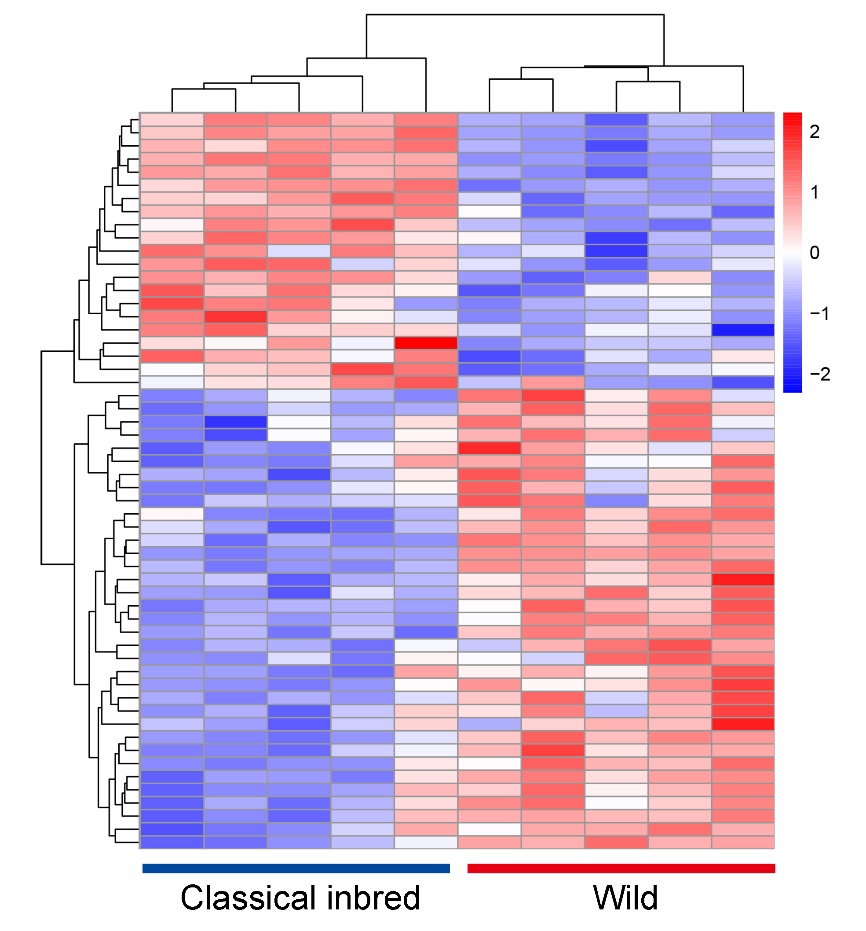
**

**Fig. S10.** The heatmap of the 56 common differently expressed genes (merged from hippocampus and frontal lobe) in the frontal lobe of mice.

**
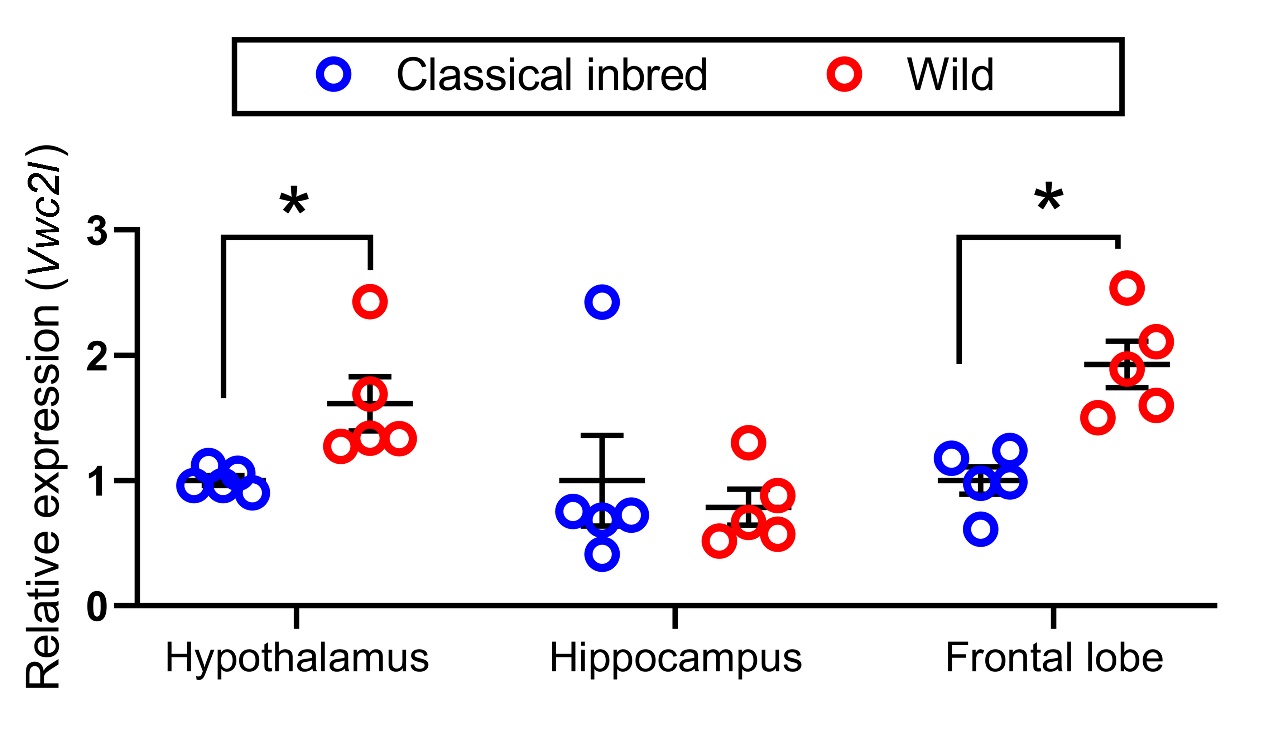
**

**Fig. S11.** The differences in relative expression of *Vwc2l* between classical inbred and wild mice. Each circle indicates one individual mouse, and error bars are standard error of mean (SEM). * indicates P < 0.05.

**
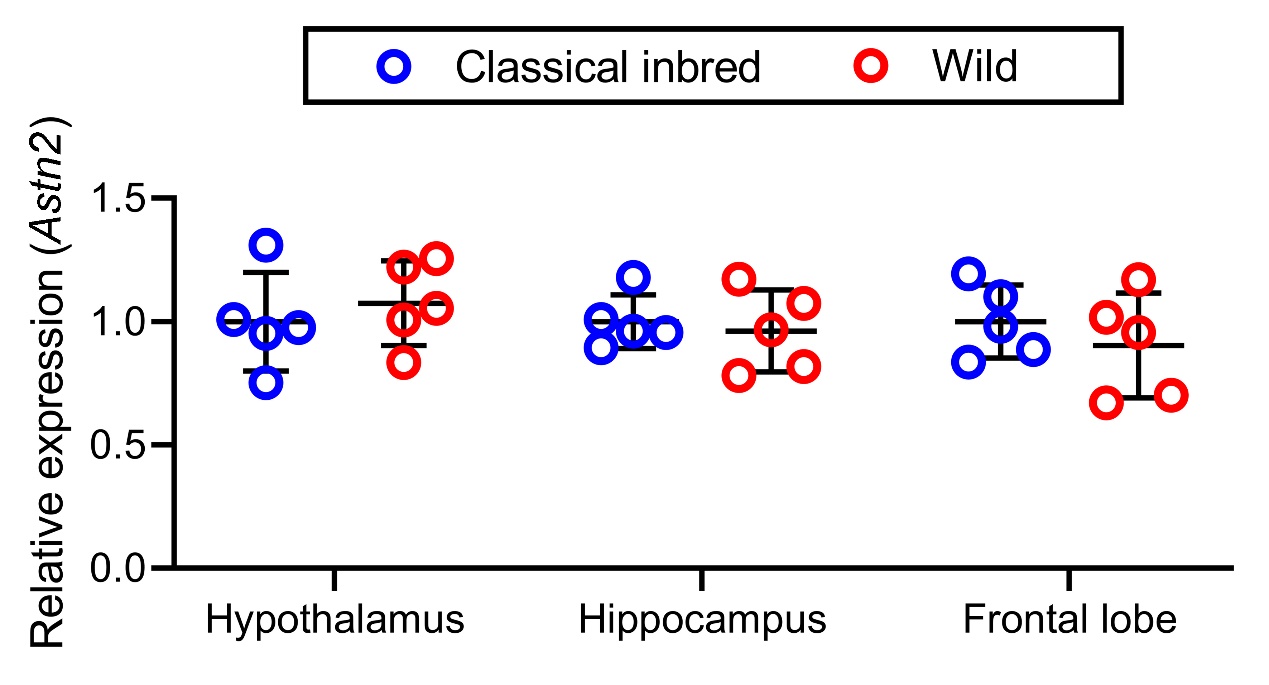
**

**Fig. S12.** The differences in relative expression of *Astn2* between classical inbred and wild mice. Each circle indicates one individual mouse, and the error bars are SEM.

**
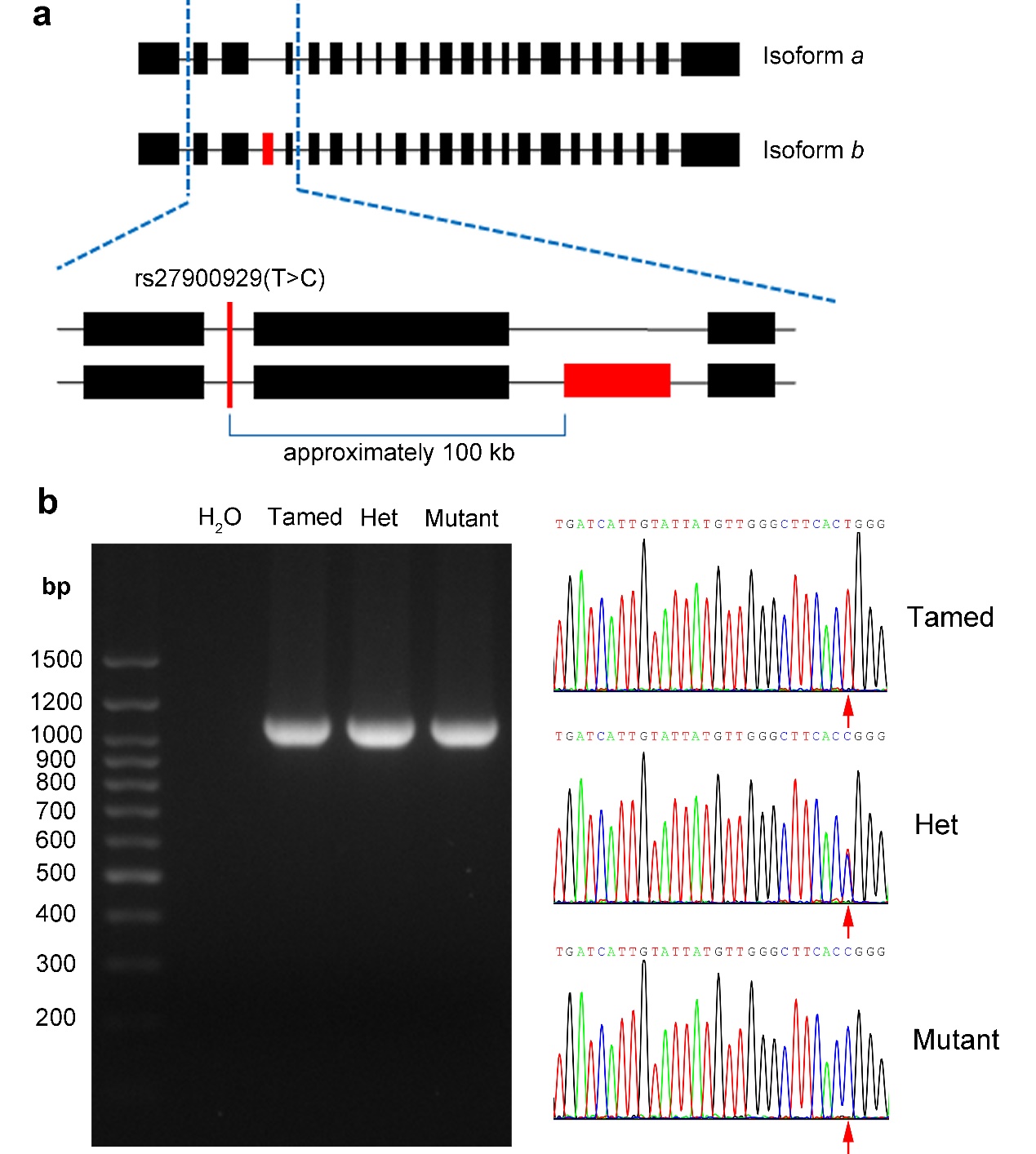
**

**Fig. S13.** The mutant mice model of rs27900929 in *Astn2* gene. a. The position of rs27900929 in the *Astn2* gene. The rectangles indicate exons of *Astn2*, and the red rectangles indicate the exon specially exist in the isoform *b*. b. The identification of *Astn2* mutant mice. The bands for sequencing is 1085 bp. Red arrow heads indicate the mutant position.

**
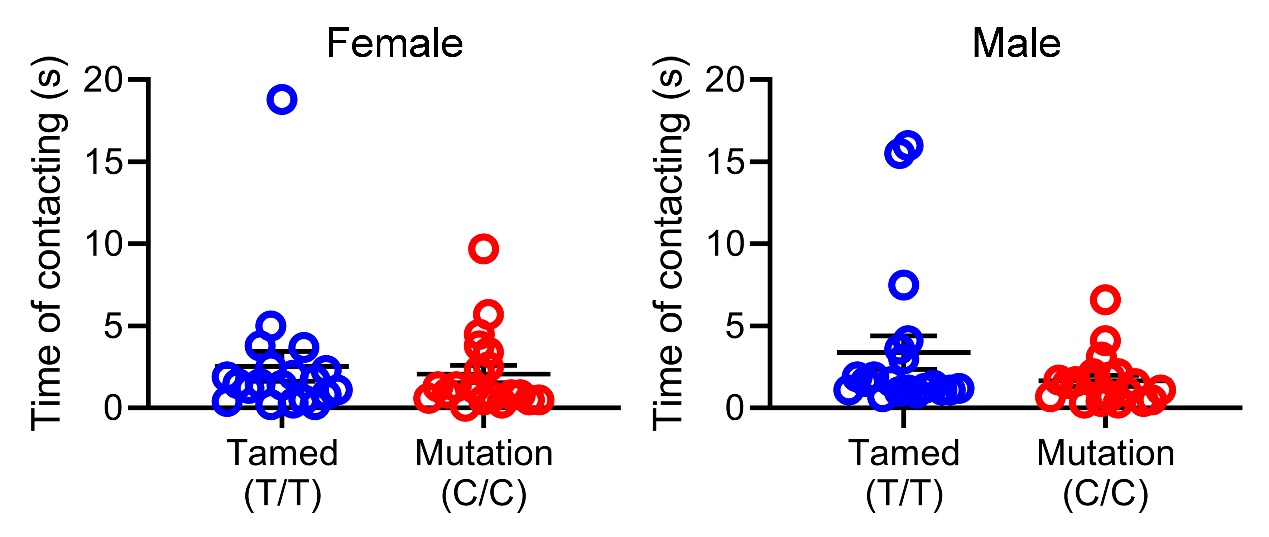
**

**Fig. S14.** The differences in active tameness (actively contacting the hand of operators) between the tamed and mutant mice. Each circle indicates one individual mouse, and error bars are SEM.

**
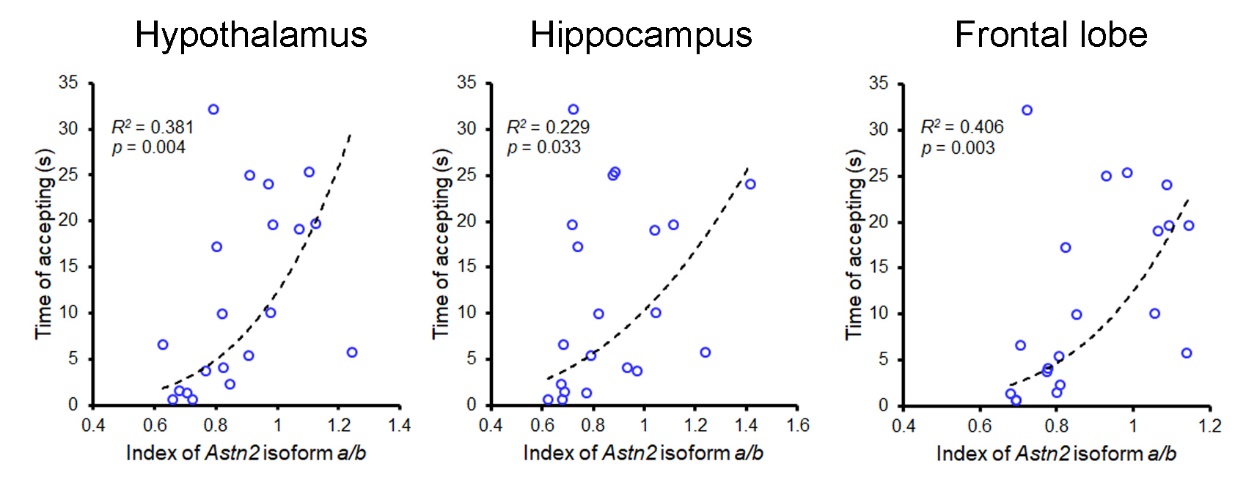
**

**Fig. S15.** The exponential relationship between accepting time and ratio of *Astn2* isoform *a/b*. Each circle indicates one individual mouse. Passive tameness was the tolerance of the animal to the touch from a human hand, as measured by accepting time.

**
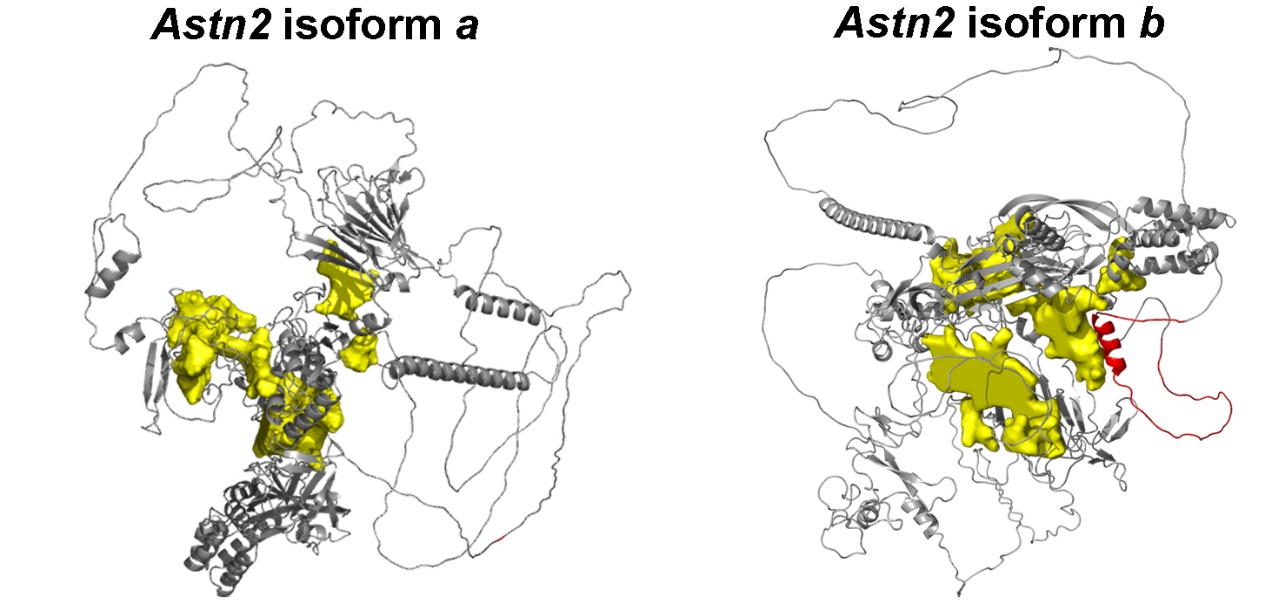
**

**Fig. S16.** The binding pockets of the proteins of the *Astn2* isoform *a* and *b*. The red color indicates the different area (Exon 4), and the yellow color indicates the large pockets. Isoform *a* lacks an alpha helix, and there is a binding pocket nearby the alpha helix of isoform *b*.


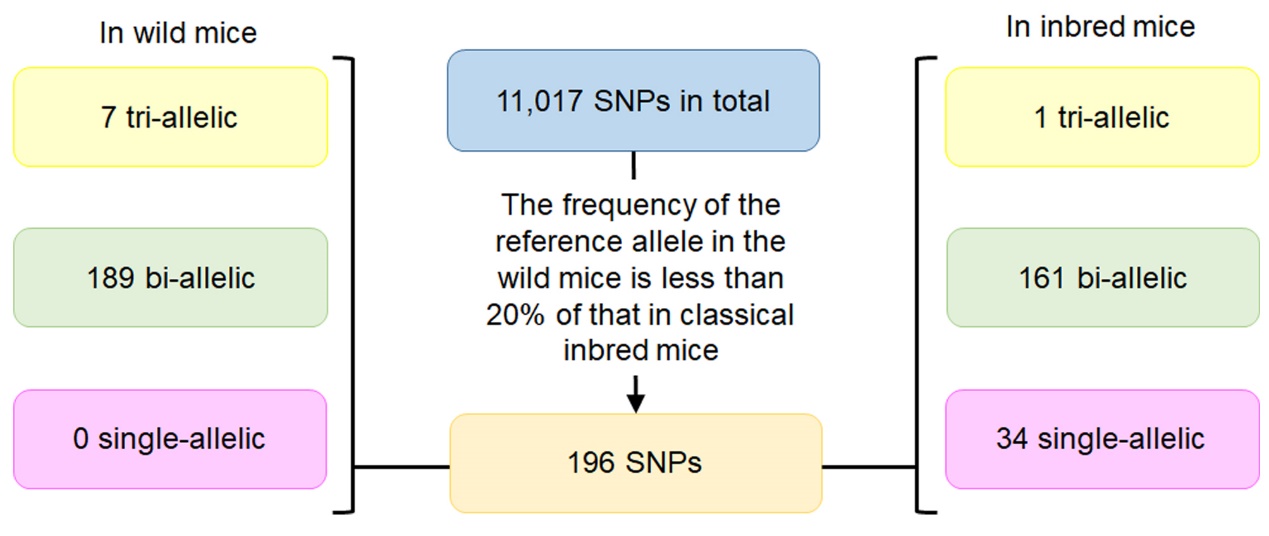


**Fig. S17.** The frequency and their relationship of tri-, bi- and single allele of SNPs of *Astn2* in wild and classical inbred mice by using the criterion that the frequency of the reference allele in the wild mice is less than 20% of that in classical inbred mice.

| **Table S1.** The characters of the 36 wild mouse samples. | | | | |
| --- | --- | --- | --- | --- |
| **Number** | **Sample name** | **Subspecies** | **Gender** | **Places of capture** |
| 1 | JK3 | *M. m. domesticus* | Male | Sendenhorst, Germany |
| 2 | JK4 | *M. m. domesticus* | Female | Sendenhorst, Germany |
| 3 | JK7 | *M. m. domesticus* | Male | Sendenhorst, Germany |
| 4 | JPC2705 | *M. m. domesticus* | Female | Straas, Germany |
| 5 | JPC2711 | *M. m. domesticus* | Male | Straas, Germany |
| 6 | JPC2716 | *M. m. domesticus* | Male | Straas, Germany |
| 7 | JPC2788 | *M. m. domesticus* | Female | Straas, Germany |
| 8 | SINJ | *M. m. domesticus* | Male | Sinj, Croatia |
| 9 | SIT | *M. m. domesticus* | Male | The Orkney Islands, Scotland, UK |
| 10 | SPOS | *M. m. domesticus* | Male | Migiondo, Italy |
| 11 | SU620 | *M. m. domesticus* | Female | Schweben, Germany |
| 12 | JCP2851 | *M. m. musculus* | Female | Buskovice, Czech Republic |
| 13 | SK822 | *M. m. musculus* | Female | Tarnowiec, Poland |
| 14 | SU5218 | *M. m. musculus* | Female | Peski Shkili, Russia |
| 15 | HB1 | *M. m. musculus* | Male | Harbin, China |
| 16 | HB2 | *M. m. musculus* | Male | Harbin, China |
| 17 | HB3 | *M. m. musculus* | Male | Harbin, China |
| 18 | ZZ1 | *M. m. musculus* | Male | Zhengzhou, China |
| 19 | ZZ2 | *M. m. musculus* | Male | Zhengzhou, China |
| 20 | ZZ3 | *M. m. musculus* | Male | Zhengzhou, China |
| 21 | WH1 | *M. m. castaneus* | Female | Wuhan, China |
| 22 | WH2 | *M. m. castaneus* | Female | Wuhan, China |
| 23 | WH3 | *M. m. castaneus* | Female | Wuhan, China |
| 24 | WH4 | *M. m. castaneus* | Female | Wuhan, China |
| 25 | WH5 | *M. m. castaneus* | Female | Wuhan, China |
| 26 | WH6 | *M. m. castaneus* | Female | Wuhan, China |
| 27 | WH7 | *M. m. castaneus* | Male | Wuhan, China |
| 28 | WH8 | *M. m. castaneus* | Female | Wuhan, China |
| 29 | WH9 | *M. m. castaneus* | Female | Wuhan, China |
| 30 | WH10 | *M. m. castaneus* | Female | Wuhan, China |
| 31 | CS1 | *M. m. castaneus* | Male | Changsha, China |
| 32 | CS2 | *M. m. castaneus* | Male | Changsha, China |
| 33 | CS3 | *M. m. castaneus* | Male | Changsha, China |
| 34 | CS4 | *M. m. castaneus* | Female | Changsha, China |
| 35 | CS5 | *M. m. castaneus* | Male | Changsha, China |
| 36 | CS6 | *M. m. castaneus* | Female | Changsha, China |

| **Table S2.** The characters of the 36 inbred mouse strains downloaded from Sanger Institute. | | | | |
| --- | --- | --- | --- | --- |
| **Number** | **Strain name** | **Type** | **Genders** | **Depth** |
| 1 | 129P2/OlaHsd | Classical inbred | Female | 52 |
| 2 | 129S1/SvImJ | Classical inbred | Female | 68 |
| 3 | 129S5SvEvBrd | Classical inbred | Female | 22 |
| 4 | A/J | Classical inbred | Female | 52 |
| 5 | AKR/J | Classical inbred | Female | 57 |
| 6 | BALB/cJ | Classical inbred | Female | 62 |
| 7 | BTBR T+ Itpr3tf/J | Classical inbred | Male | 85 |
| 8 | BUB/BnJ | Classical inbred | Male | 49 |
| 9 | C3H/HeH | Classical inbred | Female | 14 |
| 10 | C3H/HeJ | Classical inbred | Female | 63 |
| 11 | C57BL/10J | Classical inbred | Male | 37 |
| 12 | C57BL/6NJ | Classical inbred | Female | 61 |
| 13 | C57BR/cdJ | Classical inbred | Male | 51 |
| 14 | C57L/J | Classical inbred | Male | 64 |
| 15 | C58/J | Classical inbred | Male | 55 |
| 16 | CAST/EiJ | Wild-derived inbred | Female | 53 |
| 17 | CBA/J | Classical inbred | Female | 56 |
| 18 | DBA/1J | Classical inbred | Male | 49 |
| 19 | DBA/2J | Classical inbred | Female | 56 |
| 20 | FVB/NJ | Classical inbred | Female | 73 |
| 21 | I/LnJ | Classical inbred | Male | 45 |
| 22 | KK/HiJ | Classical inbred | Male | 55 |
| 23 | LEWES/EiJ | Wild-derived inbred | Female | 19 |
| 24 | LP/J | Classical inbred | Female | 54 |
| 25 | MOLF/EiJ | Wild-derived inbred | Male | 40 |
| 26 | NOD/ShiLtJ | Classical inbred | Female | 66 |
| 27 | NZB/B1NJ | Classical inbred | Male | 47 |
| 28 | NZO/HlLtJ | Classical inbred | Female | 72 |
| 29 | NZW/LacJ | Classical inbred | Male | 58 |
| 30 | PWK/PhJ | Wild-derived inbred | Female | 53 |
| 31 | RF/J | Classical inbred | Male | 54 |
| 32 | SEA/GnJ | Classical inbred | Male | 49 |
| 33 | SPRET/EiJ | Wild-derived inbred | Female | 67 |
| 34 | ST/bJ | Classical inbred | Male | 81 |
| 35 | WSB/EiJ | Wild-derived inbred | Female | 51 |
| 36 | ZALENDE/EiJ | Wild-derived inbred | Male | 19 |

| **Table S3.** The sequencing characters of the 36 wild mouse samples. | | | | | |
| --- | --- | --- | --- | --- | --- |
| **Number** | **Sample** | **Reads number (M)** | **Bases**  **(G bps)** | **Depth** | **Coverage**  **(%)** |
| 1 | JK3 | 338.4 | 34.2 | 12.5 | 95.3 |
| 2 | JK4 | 326.3 | 33.0 | 12.1 | 93.3 |
| 3 | JK7 | 324.8 | 32.8 | 12.0 | 95.1 |
| 4 | JPC2705 | 309.0 | 31.2 | 11.5 | 93.2 |
| 5 | JPC2711 | 305.3 | 30.8 | 11.3 | 94.9 |
| 6 | JCP2716 | 327.4 | 33.1 | 12.1 | 95.0 |
| 7 | JPC2788 | 297.7 | 30.1 | 11.0 | 93.3 |
| 8 | SINJ | 483.6 | 48.8 | 17.9 | 95.4 |
| 9 | SIT | 412.9 | 41.7 | 15.3 | 95.3 |
| 10 | SPOS | 416.0 | 42.0 | 15.4 | 95.2 |
| 11 | SU620 | 302.4 | 30.5 | 11.2 | 93.3 |
| 12 | JCP2851 | 462.2 | 46.7 | 17.1 | 94.8 |
| 13 | SK822 | 241.8 | 24.4 | 9.0 | 91.6 |
| 14 | SU5218 | 295.8 | 29.9 | 11.0 | 91.9 |
| 15 | HB1 | 375.8 | 38.0 | 13.9 | 94.8 |
| 16 | HB2 | 441.5 | 44.6 | 16.4 | 94.9 |
| 17 | HB3 | 450.6 | 45.5 | 16.7 | 95.0 |
| 18 | ZZ1 | 354.4 | 35.8 | 13.1 | 95.0 |
| 19 | ZZ2 | 327.3 | 33.1 | 12.1 | 95.0 |
| 20 | ZZ3 | 423.4 | 42.8 | 15.7 | 95.2 |
| 21 | WH1 | 260.9 | 39.4 | 14.5 | 94.6 |
| 22 | WH2 | 276.0 | 41.7 | 15.3 | 94.6 |
| 23 | WH3 | 373.1 | 56.3 | 20.7 | 94.4 |
| 24 | WH4 | 253.8 | 38.3 | 14.1 | 92.0 |
| 25 | WH5 | 326.1 | 49.2 | 18.1 | 94.5 |
| 26 | WH6 | 298.4 | 45.1 | 16.5 | 91.9 |
| 27 | WH7 | 260.0 | 39.3 | 14.4 | 91.7 |
| 28 | WH8 | 281.7 | 42.5 | 15.6 | 91.7 |
| 29 | WH9 | 255.8 | 38.6 | 14.2 | 91.9 |
| 30 | WH10 | 228.4 | 34.5 | 12.7 | 92.1 |
| 31 | CS1 | 287.0 | 43.3 | 15.9 | 92.1 |
| 32 | CS2 | 288.7 | 43.6 | 16.0 | 92.2 |
| 33 | CS3 | 258.0 | 39.0 | 14.3 | 91.9 |
| 34 | CS4 | 292.6 | 44.2 | 16.2 | 94.2 |
| 35 | CS5 | 267.8 | 40.4 | 14.8 | 92.1 |
| 36 | CS6 | 246.9 | 37.3 | 13.7 | 91.9 |

| **Table S4.** Number of raw SNPs and their distributions in wild and classical inbred mice*. | | | | | |
| --- | --- | --- | --- | --- | --- |
| **Number** | **Sample name** | **Total SNPs** | **Exonic** | **Intronic** | **Intergenic** |
| 1 | *M. m. domesticus* | 17,295,344 | 143,421 | 39,97,285 | 9,949,066 |
| 2 | *M. m. musculus* | 29,740,023 | 227,146 | 69,89,778 | 17,042,733 |
| 3 | *M. m. castaneus* | 38,325,000 | 269,586 | 9,045,198 | 21,931,641 |
| 4 | Classical inbred | 12,505,187 | 100,276 | 3,130,111 | 7,051,985 |

* The number of total SNPs is larger than the sum of exonic, intronic, and intergenic SNP number, because a part of SNPs locates at the non-coding gene region and gene UTRs.

| **Table S5.** Number of raw SNPs and their distributions in wild-derived inbred mice originating from *M. musculus**. | | | | | |
| --- | --- | --- | --- | --- | --- |
| **Number** | **Sample name** | **Total SNPs** | **Exonic** | **Intronic** | **Intergenic** |
| 1 | LEWES/EiJ | 4,884,269 | 40,566 | 1,224,039 | 2,771,707 |
| 2 | WSB/EiJ | 4,903,673 | 40,633 | 1,219,480 | 2,800,708 |
| 3 | ZALENDE/EiJ | 5,603,599 | 45,632 | 1,415,474 | 3,178,706 |
| 4 | PWK/PhJ | 14,757,431 | 107,949 | 3,751,005 | 8,231,316 |
| 5 | MOLF/EiJ | 14,203,889 | 104,570 | 3,644,697 | 7,967,583 |
| 6 | CAST/EiJ | 15,091,063 | 109,541 | 3,854,025 | 8,452,959 |

* The number of total SNPs is larger than the sum of exonic, intronic, and intergenic SNP number, because a part of SNPs locates at the non-coding gene region and gene UTRs.

| **Table S6.** Wild-derived inbred mice and their wild relatives. | | |
| --- | --- | --- |
| **Number** | **Mice strains** | **Ancestor reported previously** |
| 1 | CAST/EiJ | *M. m. castaneus* |
| 2 | LEWES/EiJ | *M. m. domesticus* |
| 3 | MOLF/EiJ | *M. m. molossinus** |
| 4 | PWK/PhJ | *M. m. musculus* |
| 5 | WSB/EiJ | *M. m. domesticus* |
| 6 | ZALENDE/EiJ | *M. m. domesticus* |
| * *M. m. molossinus* is the hybrids between *M. m. castaneus* and *M. m. musculus*. | | |

**Table S21.** Details of the tameness test in tamed and mutant mice.

|  |  | **Tamed (T/T)** | | **Mutation (C/C)** | ***p* value** |
| --- | --- | --- | --- | --- | --- |
| **Female** | Active contacting time (s) | 2.54 ± 4.04 | | 2.07 ± 2.38 | 0.657 |
|  | Passive accepting time (s) | 16.46 ± 8.22 | | 5.31 ± 7.67 | <0.001 |
| **Male** | Active contacting time (s) | 3.38 ± 4.52 | | 1.66 ± 1.52 | 0.121 |
|  | Passive accepting time (s) | 20.10 ± 9.88 | | 4.98 ± 6.21 | <0.001 |
|  | Biting (n) | 0.05 ± 0.22 | | 0.55 ± 0.76 | 0.01 |
| **Pearson's correlation of the measurements** | | | ***R* value** | | ***p* value** |
| Active contacting Vs Passive accepting | | | 0.300 | | 0.07 |
| Active contacting Vs Biting | | | 0.109 | | 0.502 |
| Passive accepting Vs Biting | | | 0.399 | | 0.01 |

**Table S23.** Constructed mouse models for tameness test*.

| **Number** | **Gene** | **Position** | **Mutation** | **Tameness modification** |
| --- | --- | --- | --- | --- |
| 1 | *Sqstm1* | Chr11: 50200767 | G > GGTGGGTC | No |
| 2 | *Astn2* | Chr4: 66226438 | T > C | Yes |
| 3 | *Astn2* | Chr4: 66221175 | A > T | No |
| 4 | *Astn2* | Chr4: 65760056 | G > A | Inadequate sample size |
| 5 | *Eea1* | Chr10: 95959755 | C > A | Inadequate sample size |

*: *Sqstm1* is not relevant to this study. It is constructed together with the four models involved in this study.

**Table S26.** Primers used in this study.

| **Number** | **Gene** | **F-primer** | **R-primer** | **Other information** |
| --- | --- | --- | --- | --- |
| 1 | *Astn2* | TCCCAGTTTTCTTCCGTGATG | AGTGCCCTGGTTTTATTGTTTATG | For the identification *Astn2* mutant mice |
| 2 | *Astn2* | CCCGTGAGGATGAGTTTGGT | GCTGATTCCCCCTTTCTTCTCA | For qPCR of total *Astn2* |
| 3 | *Astn2* | AAGAGGAGGAGGAACCACCC | CTTCTGAGCCTGATGTCCCC | For qPCR of *Astn2* isoform *a* |
| 4 | *Astn2* | TGGAGATCGGTCAGTTGCAG | CTTCTGAGCCTGATGTCCCC | For qPCR of *Astn2* isoform *b* |
| 5 | *Kcnd2* | TCGTGTCGAACTTCAGTCGG | TTGCTCAGTAGCCCATTCCG | For qPCR of *Kcnd2* |
| 6 | *Sebox* | AATCGGCGAGCCAAGAGAAT | ACTGGTAGGGTGCAGAGGAT | For qPCR of *Sebox* |
| 7 | *Vwc2l* | AGGAATTTAAGGTCCAAACTGCT | CTTGGCATTCGCGCTTTGAA | For qPCR of *Vwc2l* |
| 8 | *Gapdh* | GGAGAAACCTGCCAAGTATGATG | AAGAGTGGGAGTTGCTGTTGAAG | For qPCR of *Gapdh* (ref gene) |
