# Supplementary material for: Whole-genome sequencing reveals the genetic mechanisms of domestication in classical inbred mice
Source: Genome Biol. 2022 Sep 26;23:203. doi: 10.1186/s13059-022-02772-1 (PMC9511766; doi:10.1186/s13059-022-02772-1)
Supplement: Supplementary file 6 — Additional file 6. Review history. [file 13059_2022_2772_MOESM6_ESM.docx]

**Review history**

**First round of review**

**Reviewer 1**

The authors have performed a detailed whole genome scan of wild and inbred mice to identify genes that have been under selection during domestication of inbred lab strains. The authors have performed a thorough analyses using different approaches and followed up their findings with RNAseq expression gene editing experiments. The paper confirms earlier findings of domestication in other species and presents interesting results regarding the effect of a mutation in the Asn2 gene on behaviour in mice. However, there are some aspects that need to be addressed by the authors. First, I think the manuscript needs to be improved for the English language used, in particular the abstract. Below under "Suggested changes to improve the text" I have made several suggestions to improve the language and making the paper clearer.

I am confused by the study to identify the most likely SNP within Asn2 as described in lines 325-345. In particular criteria 1 and 2 don't seem to make much sense to me. Why did you not use an extended haplotype homozygosity based approach like e.g. XP-EHH ( e.g. Gautier and Vitalis. (2012) rehh: a package to detect footprints of selection in genome-wide SNP data from haplotype structure Bioinformatics 28:1176; Tang, et al. (2007) A new approach for using genome scans to detect recent positive selection in the human genome. PLoS Biol. 5:1587). I also don't see how you can reach the conclusion stated in lines 344-345 that this is not a founder effect. Also how do the results exclude drift effects. This needs to be discussed more clearly.

Although the link between behaviour and a possible splicing effect of SNP rs27900929 is very interesting, I don't think the authors can completely rule out an effect on transcription levels. The fact that no differences are observed in. the level of transcription in their study does not rule out differences at other time points during development or differences in specific cell types. To study this would require time series and/or single cell transcriptome studies. These are clearly beyond the scope of the current manuscript but would need to be briefly discussed.

The grouping of the classical inbred mouse strains (Figure 1a) in addition to selection is most likely the result of the admixture with the other mouse sub-species (line 190-193 and line 417).

You should describe the effect on the protein for the two splice variants for Asn2. I assume the presence/absence of exon 4 does affect the protein.


Minor comments:
Line 203-206: Rather obvious that inbred mice exhibited severe bottlenecks, that is inherent to generating inbred lines. The same is true for founder effects. Also, the authors should better discuss drift effects in such lines.

Line 344-345: I don't see how you can conclude this from your results. You also do not discuss how you can rule out drift.

Line 348: I don't think you can refer to this mutant as a "wild-like" mutant mouse strain.

Line 351-353 seems to contradict the previous sentence. I assume the authors intent to describe "passive tameness" but it looks like it is related to "the mutant mice showed a significant decrease (60-70%) of tameness"


Suggested changes to improve the text:
Line 69-70: The laboratory mouse, an important experimental animal, was domesticated from the wild house mouse.

Line 70: Understanding the genetics underlying domestication in laboratory mice,

Line 72-74: However, the genetics of laboratory mouse domestication remains unknown due to lack of adequate genome sequences of wild mice.

Line76-77: We analyzed the genetic relationships by whole-genome resequencing of 36 wild mice and 36 inbred strains

Line 77-78: All classical inbred mice clustered together distinctly from the wild and wild-derived inbred mice

Line 80: replace "which is" by "that are"

Line 81-82: "and 125 genes exhibiting abnormal phenotypes". Not correct, not the genes exhibit a phenotype but the mice with specific variants in these genes.

Line 83-85: These PSGs showed a higher ratio of differential expression between wild and classical inbred mice as compared with all genes, especially in hippocampus and frontal lobe.

Line 85: Insert "a" before mutant

Line 114-115: Laboratory inbred mice strains are used world-wide as animal models and can be classified into two groups,

Line 153: Delete "were"

Line 154: Insert "to" before "the mouse"

Line 193-194: The clustering of classical inbred strains with the wild M. m. domesticus

Line 200: Insert "the" before "genetic background"

Line 233: Replace "were" by "are"

Line 287-289: of which the expression is approximately 50% higher in the brain tissues of wild mice

Line 295: Replace "The mutations" by "Mutations"

Line 296-298: All this evidence suggests that Kcnd2 is essential for the function of the nervous system and is involved changes of the behavior as a result from domestication of mice.

Line 302-303: and changes in behavior during the domestication process.

Line 304: Some genes shown to be only differentially expressed

Line 311: Replace "in" by "during"

Line 318: A previous study showed that double knockout of exon 5 of Astn2 and Fz6 (Frizzled6) led to a 180-degree hair orientation reversal on the back in mice [61]. However, no behavioral phenotype of Astn2 has ever been found in mutant or knockout mouse models.

Line 325: Insert "the" before Astn2.

Line 340-342: The frequency of the reference allele (T) of this SNP is only 4.17% in the wild mice, while the frequencies of the other 2 alleles (C and A) are 50.0% and 45.8%, respectively.

Line 348: constructed

Line 365: Change "was" by "is"

Line 388" Replace "locus" by "loci"

Line 401-403: We also found a different mechanism for SNP rs27900929 in gene Astn2 which affected tameness by changing the ratio of different alternative splicing variants

Line 412: Change "proved" by "proven"

Line 431: Insert "A" before mutant

**Reviewer 2**

This is a very strong study looking at the genetic effects of domestication in regard to tameness. The selective sweep aspect of the study is solid, but what elevates it is the testing of one of the candidate genes using CRISPR to validate the gene has an effect on behaviour. This makes it a very strong study investigating the genetic basis of domestication effects in mice.

1. The selective sweep comparison used the 36 wild mice and the mice genomes of lab strains that were derived from wild mice. I am unsure how valid the grouping of wild mice with these inbred strains is. The lab strains are highly inbred, and whilst they once were derived from (a very limited number) of wild mice, they have subsequently been highly inbred with large shifts in allele frequency and founder effects. To ensure that these mice are not having a disproportionate effect on the sweep scans, it would be beneficial to repeat the selective sweep analysis with just the actual wild mice as the test group.
2. The top 5% ranked genes were retained during the sweep analysis, however this seems to be a fairly arbitrary cut-off threshold. Would an FDR test or similar be more precise to use? Or at least a justification of why simply taking the top 5% of genes is viable.
3. I found it interesting that GABA-ergic synapses were an enriched gene class - this mirrors some of the domestication genes identified between wild and domestic chickens (the GABRB2 gene - see Johnsson et al. 2016. Genetics, 202, pp327-340).
4. For the relative expression of genes identified in sweep regions (lines 244 onwards), without doing tests, it is hard to know if the larger number of genes highly expressed in the brain is significant. There is always a great deal of gene expression in the brain, so this could easily be non-significant. Some testing whereby the relative expression of a random subset of genes would make a nice comparison to be able to actually derive a proper significance test.
5. For the tissue comparison (see lines 284 and onwards) in PSGs, common genes that were identified in two of the brain regions were selected (hippocampus and frontal lobe), however a third (and very valid) tissue was also measured - the hypothalamus. Why was this region not also included to identify these genes. Was there a good reason for omitting it from the analysis?
6. The most noteworthy aspect to this study is definitely the testing of a specific mutation identified in the sweep scan. The results from this represent a great stride in proving that this mutation effects behaviour. What is a little puzzling though is how this gene (or rather how this mutation) was selected for further analysis. The authors state that there was no difference in gene expression between the mutant and wild type alleles, yet numerous of the other genes that were detected with different expression levels between the morphs. In this case, surely this gene would have been much lower down the list of potential candidates. Were more genes/ alleles tested as well? The rationale for the selection of candidate SNPs (tri-allelic in wild, bi-allelic in inbred, 20% reduced frequency in the wild population) is not really expanded upon. I.e. why a 20% drop in frequency, why tri-allelic SNPs only. What I think is really lacking is the justification for the selection of Astn2. Of course, the final results of the CRISPR seem very convincing, but even if more mutations were tested but found to have no effect, these should still be mentioned and are important data.
7. With the CRIPSR effects, how does the decrease in the proportions of the two isoforms compare to the change in behaviour? I am just wondering if the reduction in fear behaviour mirrors the 20-30% drop seen in the allele isoforms (I,e. a 20% shift in isoform also leads to a 20% shift in behaviour). When discussing the isoform effects, the authors should also note that the mutation tested could also be a trans acting effect, and the isoform alterations are merely coincidental. No expression evidence for example shows that the Astn2 gene is causal (though good proof exists for the SNP itself).
8. Some more details on the behavioural test used to assay the transgenic mice is required. How many parameters were measured? What were the populations metrics (means, standard deviations, etc) of the traits tested, what was the relationship between passive and active tameness measures (and indeed all measurements taken)? Similarly, what were the p-values for the tests between the strains, differences between strains in terms of standard deviations, etc.
9. The English language is good, but there are quite a few incidents of grammatical errors that pop up fairly often. I should stress that these are not so severe that they really disrupt the flow of reading, but they would certainly be good to address. For example (not exhaustive), line 563 "to avoid hurts of the mouse", line 114 "were the world-widely used animal model", line 354 "Approximately 30% the male mutant individuals".

**Authors’ response to reviewers**

Reviewer #1:
The authors have performed a detailed whole genome scan of wild and inbred mice to identify genes that have been under selection during domestication of inbred lab strains. The authors have performed a thorough analyses using different approaches and followed up their findings with RNAseq expression gene editing experiments. The paper confirms earlier findings of domestication in other species and presents interesting results regarding the effect of a mutation in the Asn2 gene on behaviour in mice. However, there are some aspects that need to be addressed by the authors.

First, I think the manuscript needs to be improved for the English language used, in particular the abstract. Below under "Suggested changes to improve the text" I have made several suggestions to improve the language and making the paper clearer.

Response: We asked a native English speaker to improve the English language.

I am confused by the study to identify the most likely SNP within Asn2 as described in lines 325-345. In particular criteria 1 and 2 don't seem to make much sense to me.

Response: Sorry for the unclear description. Criteria 1 and 2 were used to minimize the effects of genetic drift or founder effect, which is reflected by the low genetic diversity. The first criterion was that the SNPs should have at least three alleles in the wild mice, which aimed to ensure high SNP diversity in the wild population. The second criterion is that SNPs should have at least two alleles in inbred mice, which aimed to minimize the genetic drift and founder effect, since a single allele in classical inbred population may be more likely caused by genetic drift or founder effect. The third criterion is that the frequency of the specific allele in the wild mice should be less than 20% of that in classical inbred mice, aiming to increase the probability of selecting the allele under artificial selection of inbred mice. In summary, our three criteria aimed to minimize the influence of genetic drift or founder effect from wild mice to classical inbred mice, and to select a specific allele with a higher probability under artificial selection. We modified these sentences (line 330-343, 351-353).

Why did you not use an extended haplotype homozygosity based approach like e.g. XP-EHH (e.g. Gautier and Vitalis. (2012) rehh: a package to detect footprints of selection in genome-wide SNP data from haplotype structure Bioinformatics 28:1176; Tang, et al. (2007) A new approach for using genome scans to detect recent positive selection in the human genome. PLoS Biol. 5:1587).

Response: We identified the selected genes from the whole genome by using nucleotide diversity, Fst, and XP-CLR. XP-CLR is an analogy to the extended haplotype homozygosity test (Chen et al. Genome Res. 2010;20(3):393-402), which was used as a dependent algorithm with XP-EHH. A previous study showed that XP-CLR exhibited a higher power to detect the selected sweeps and to exclude gene drift than XP-EHH (Vatsiou et al. Mol Ecol. 2016;25(1):89-103 [PMID: 26314386]). We highlighted the XP-CLR is a dependent algorithm with XP-EHH in the method (line 537-538)

I also don't see how you can reach the conclusion stated in lines 344-345 that this is not a founder effect. Also how do the results exclude drift effects. This needs to be discussed more clearly.

Response: Sorry for this incorrect description. We modified the sentence. We have clarified how we minimized the effect of genetic drift or founder effect in the Methods section (line 330-343, 351-353).

Although the link between behaviour and a possible splicing effect of SNP rs27900929 is very interesting, I don't think the authors can completely rule out an effect on transcription levels. The fact that no differences are observed in. the level of transcription in their study does not rule out differences at other time points during development or differences in specific cell types. To study this would require time series and/or single cell transcriptome studies. These are clearly beyond the scope of the current manuscript but would need to be briefly discussed.

Response: Thank you for pointing out this. Indeed, the SNP rs27900929 could influence the Astn2 gene expression in developing stage or in a special type of cell, and finally affecting behavior of mice. We added such statement in the manuscript (line 368-374).

The grouping of the classical inbred mouse strains (Figure 1a) in addition to selection is most likely the result of the admixture with the other mouse sub-species (line 190-193 and line 417).

Response: We agree with you on this. Indeed, the genomes of inbred mice strains are the mosaic of the three subspecies apart from artificial selection. We modified the description (line 191-192; line 454-455).

You should describe the effect on the protein for the two splice variants for Asn2. I assume the presence/absence of exon 4 does affect the protein.

Response: There is no previous report or study on the functional change between the two splice variants of Astn2. Indeed, the lack of exon 4 did not cause frameshift in Astn2 isoform b. However, prediction using AlphaFold2 and other analyses showed that the structure and binding pockets of the 2 isoforms had obvious difference, indicating there may be functional difference between the two splice variants (Additional file 1: Fig. S17). We added results of additional analysis and the relevant discussion in the revised version (line 385-388; 430-437).

Minor comments:
Line 203-206: Rather obvious that inbred mice exhibited severe bottlenecks, that is inherent to generating inbred lines. The same is true for founder effects. Also, the authors should better discuss drift effects in such lines.

Response: We added sentences to discuss genetic drift and founder effect in the discussion (line 203-209).

Line 344-345: I don't see how you can conclude this from your results. You also do not discuss how you can rule out drift.

Response: Sorry for the unclear description. We have clarified this in methods by giving a clear explanation on how we minimized the genetic drift or founder effect (line 330-343, 351-353).

Line 348: I don't think you can refer to this mutant as a "wild-like" mutant mouse strain.

Response: We deleted the word (line 356).

Line 351-353 seems to contradict the previous sentence. I assume the authors intent to describe "passive tameness" but it looks like it is related to "the mutant mice showed a significant decrease (60-70%) of tameness"

Response: We changed the sentence to “the mutant mice showed a significant decrease (60–70%) of passive tameness as measured by accepting time (i.e. tolerance time) to the touch of human hand [57]. In other words, the mutant mice often ran away or stretched their body to avoid the touch of human fingertips (Fig. 4c, 4d, Additional file 3: Video S1 and Additional file 4: Video S2)” (line 358-362).

Suggested changes to improve the text:
Line 69-70: The laboratory mouse, an important experimental animal, was domesticated from the wild house mouse. Corrected (line 69-70)
Line 70: Understanding the genetics underlying domestication in laboratory mice, Corrected (line 70)
Line 72-74: However, the genetics of laboratory mouse domestication remains unknown due to lack of adequate genome sequences of wild mice. Corrected (line 72-73)
Line76-77: We analyzed the genetic relationships by whole-genome resequencing of 36 wild mice and 36 inbred strains. Corrected (line 75-76)
Line 77-78: All classical inbred mice clustered together distinctly from the wild and wild-derived inbred mice. Corrected (line 76-77)
Line 80: replace "which is" by "that are". Corrected (line 78)
Line 81-82: "and 125 genes exhibiting abnormal phenotypes". Not correct, not the genes exhibit a phenotype but the mice with specific variants in these genes. Corrected (line 80-81)
Line 83-85: These PSGs showed a higher ratio of differential expression between wild and classical inbred mice as compared with all genes, especially in hippocampus and frontal lobe. Corrected (line 81-83)
Line 85: Insert "a" before mutant. Corrected (line 83)
Line 114-115: Laboratory inbred mice strains are used world-wide as animal models and can be classified into two groups, Corrected (line 113-114)
Line 153: Delete "were". Corrected (line 152)
Line 154: Insert "to" before "the mouse" Corrected (line 153)
Line 193-194: The clustering of classical inbred strains with the wild M. m. domesticus. Corrected (line 193-194)
Line 200: Insert "the" before "genetic background". Corrected (line 200)
Line 233: Replace "were" by "are". Corrected (line 235)
Line 287-289: of which the expression is approximately 50% higher in the brain tissues of wild mice. Corrected (line 301-302)
Line 295: Replace "The mutations" by "Mutations". Corrected (line 309)
Line 296-298: All this evidence suggests that Kcnd2 is essential for the function of the nervous system and is involved changes of the behavior as a result from domestication of mice. Corrected (line 310-312)
Line 302-303: and changes in behavior during the domestication process. Corrected (line 316-317)
Line 304: Some genes shown to be only differentially expressed. Corrected (line 318)
Line 311: Replace "in" by "during". Corrected (line 325)
Line 318: A previous study showed that double knockout of exon 5 of Astn2 and Fz6 (Frizzled6) led to a 180-degree hair orientation reversal on the back in mice [61]. However, no behavioral phenotype of Astn2 has ever been found in mutant or knockout mouse models. Corrected (line 413-416)
Line 325: Insert "the" before Astn2. Corrected (line 330)
Line 340-342: The frequency of the reference allele (T) of this SNP is only 4.17% in the wild mice, while the frequencies of the other 2 alleles (C and A) are 50.0% and 45.8%, respectively. Corrected (line 347-349)
Line 348: constructed. Corrected (line 356)
Line 365: Change "was" by "is". Corrected (line 378)
Line 388" Replace "locus" by "loci". Corrected (line 408)
Line 401-403: We also found a different mechanism for SNP rs27900929 in gene Astn2 which affected tameness by changing the ratio of different alternative splicing variants. Corrected (line 428-430)
Line 412: Change "proved" by "proven". Corrected (line 449)
Line 431: Insert "A" before mutant. Corrected (line 469)

Response: Many thanks for your efforts in improving the writing of our manuscript. We modified the manuscript according to your suggestions.


Reviewer #2:
This is a very strong study looking at the genetic effects of domestication in regard to tameness. The selective sweep aspect of the study is solid, but what elevates it is the testing of one of the candidate genes using CRISPR to validate the gene has an effect on behaviour. This makes it a very strong study investigating the genetic basis of domestication effects in mice.

1. The selective sweep comparison used the 36 wild mice and the mice genomes of lab strains that were derived from wild mice. I am unsure how valid the grouping of wild mice with these inbred strains is. The lab strains are highly inbred, and whilst they once were derived from (a very limited number) of wild mice, they have subsequently been highly inbred with large shifts in allele frequency and founder effects. To ensure that these mice are not having a disproportionate effect on the sweep scans, it would be beneficial to repeat the selective sweep analysis with just the actual wild mice as the test group.

Response: Thanks for this suggestion. We re-analyzed the selected genes using only the classical inbred mice and the 36 wild mice, and got 355 selected genes. The new selected gene list was very similar with the former 339 PSGs with 258 gene overlapping with those of previous analysis. We added the results of new analysis in the result section (line 246-249 and Additional file 2: Table S15).

2. The top 5% ranked genes were retained during the sweep analysis, however this seems to be a fairly arbitrary cut-off threshold. Would an FDR test or similar be more precise to use? Or at least a justification of why simply taking the top 5% of genes is viable.

Response: Sorry for unclear description. Arbitrary top 1 or 5% cut-off threshold has been widely used in the literatures (e.g. Zhou et al. Mol Biol Evol. 2016;33(10):2670-81; Yang et al. Mol Biol Evol. 2016;33(10):2576-92; Li et al. Nat Genet. 2013;45(12):1431-8; Ding et al. Nat Commun. 2018;26;9(1):4991; Nanaei et al. Genomics. 2020;112(1):880-885). In our study, we used a top 5% cut-off threshold based on the results by using 3 different filtering methods, which would allow us to have a higher chance of selecting the artificial-selected genes in inbred mice.

3. I found it interesting that GABA-ergic synapses were an enriched gene class - this mirrors some of the domestication genes identified between wild and domestic chickens (the GABRB2 gene - see Johnsson et al. 2016. Genetics, 202, pp327-340).

Response: Thank you for pointing out this link. We discussed our results with that of previous findings as to GABA-ergic synapses (line 402-404).

4. For the relative expression of genes identified in sweep regions (lines 244 onwards), without doing tests, it is hard to know if the larger number of genes highly expressed in the brain is significant. There is always a great deal of gene expression in the brain, so this could easily be non-significant. Some testing whereby the relative expression of a random subset of genes would make a nice comparison to be able to actually derive a proper significance test.

Response: Thank you for this suggestion. We re-analyzed the 286 genes included in the Mouse ENCODE transcriptome database (PRJNA66167) by using RankSum test, and found that 140 genes exhibited significant increase in immature brain/brain tissues as compared to other tissues. We added results of the new analysis in the revised version (line 258-261); and in supplementary materials (Additional file 2: Table. S16).

5. For the tissue comparison (see lines 284 and onwards) in PSGs, common genes that were identified in two of the brain regions were selected (hippocampus and frontal lobe), however a third (and very valid) tissue was also measured - the hypothalamus. Why was this region not also included to identify these genes. Was there a good reason for omitting it from the analysis?

Response: The hippocampus and frontal lobe exhibited 1.8 and 2.0 fold changes in ratio of significantly differentially expressed gene in PSGs as compared to all genes, while hypothalamus only exhibited 1.3 fold change, similar to those in the heart, liver, and lung tissues. These results indicated that hippocampus and frontal lobe should be more closely associated with nervous system functions. Thus, we only presented results of hippocampus and frontal lobe, not hypothalamus in previous version. A previous study in rats also exhibited similar results (See ref 13). We added some sentences explaining why we selected hippocampus and frontal lobe for tissue comparisons in the manuscript (line 290-297). Also, we presented the results on the differently expressed genes of the 339 PSGs in hypothalamus in supplementary materials (Additional file 2: Table S20).

6. The most noteworthy aspect to this study is definitely the testing of a specific mutation identified in the sweep scan. The results from this represent a great stride in proving that this mutation effects behaviour. What is a little puzzling though is how this gene (or rather how this mutation) was selected for further analysis. The authors state that there was no difference in gene expression between the mutant and wild type alleles, yet numerous of the other genes that were detected with different expression levels between the morphs. In this case, surely this gene would have been much lower down the list of potential candidates. Were more genes/ alleles tested as well? The rationale for the selection of candidate SNPs (tri-allelic in wild, bi-allelic in inbred, 20% reduced frequency in the wild population) is not really expanded upon. I.e. why a 20% drop in frequency, why tri-allelic SNPs only. What I think is really lacking is the justification for the selection of Astn2. Of course, the final results of the CRISPR seem very convincing, but even if more mutations were tested but found to have no effect, these should still be mentioned and are important data.

Response: Sorry for this unclear description. We did two analyses in selecting the genes and SNP sites. In the first analysis, we did not have samples of wild subspecies M. m. castaneus. We performed an analysis to select genes by using wild mice of two subspecies M. m. domesticus and M. m. musculus. We found 8 genes exhibited close relationships to mental diseases and behavior, including Prkcq, Astn2, Gm20388, Pcdh15, Eea1, Nav3, Nrxn3, and Iglc2. Thus, they were used as the candidates to construct behavioral mouse models. We constructed 5 mouse models and found only Astn2 showed significant difference in behavioral performance between wild and mutant types (Additional file 1: Table S22). We did not report the results of the other genes. Now as you suggested, we added the results of the other 4 mouse models in the supplementary materials (Additional file 1: Table S22). Later on, we succeeded to capture another wild subspecies of M. m. castaneus. Then, we re-ran the selection process by including the three wild subspecies. Besides, we found the genome sequencing quality of one individual from wild M. m. musculus was not high, and it was not included in the second analysis. We found only Astn2, Pcdh15, Nrxn3 remained in the top selected gene, and the SNP rs27900929 in Astn2 gene region was still the targeted SNP site, indicating three wild subspecies could be essential in selecting the targeted genes. The function and relation with behavior of Pcdh15 and Nrxn3 have been well studied, and were not used for constructing mouse model. Limited information is known as to the function and its relation to the behavior of Astn2, Prkcq and Eea1, which were used for constructing the mouse model. Only Astn2 was found to have behavioral difference between wild and mutated mice. We did not complete behavioral assessment of some mouse models due to inadequate sample size. We added this explanation in the Methods (line 608-639), and the results of the other tested genes in supplementary materials (Additional file 1: Table S22).
As to the rationale for the selection of candidate SNPs (tri-allelic in wild, bi-allelic in inbred, 20% reduced frequency in the wild population), we expanded our explanation in Methods. This is a similar question to one raised by the first reviewer. Criteria 1 and 2 were used to exclude the effects of genetic drift or founder effect, which is characterized by low genetic diversity. The first criterion is that the SNPs should have at least three alleles in the wild mice, aiming to ensure high SNP diversity in the wild population. The second criterion is that SNPs should have at least two alleles in inbred mice to minimize the genetic drift and founder effect, since a single allele in classical inbred population may be more likely be caused by genetic drift or founder effect. The third criterion is the frequency of the specific allele in wild mice should be less than 20% of that in classical inbred mice, aiming to increase the probability of selecting the allele under artificial selection on inbred mice. In summary, our three criteria aimed to minimize the influence of genetic drift or founder effect from wild mice to classical inbred mice, and to select a specific allele with high probability under artificial selection. We modified these sentences in the revised version (line 330-343, 351-353).

7. With the CRIPSR effects, how does the decrease in the proportions of the two isoforms compare to the change in behaviour? I am just wondering if the reduction in fear behaviour mirrors the 20-30% drop seen in the allele isoforms (I,e. a 20% shift in isoform also leads to a 20% shift in behaviour). When discussing the isoform effects, the authors should also note that the mutation tested could also be a trans acting effect, and the isoform alterations are merely coincidental. No expression evidence for example shows that the Astn2 gene is causal (though good proof exists for the SNP itself).

Response: Thank you for this suggestion. The main changed behavior was passive tameness, which was measured by the tolerance time of the animal to human touch (accepting time). Based on your suggestion, we performed a regression analysis between the ratio of the 2 Astn2 isoforms and the accepting time based on the data shown in Figure 4d & 4h in previous version. The results showed that the accepting time showed an exponential increase with ratio of the 2 isoforms, which supports your prediction (Additional file 1: Fig. S16). We added this new analysis in the supplementary material (Additional file 1: Fig. S16). We also added the results in the Result section (line 383-385).
We agree with you that the isoform alterations of Astn2 may not be the direct cause of the behavior and added some sentences to explain this in the Discussion section (line 445-448).

8. Some more details on the behavioural test used to assay the transgenic mice is required. How many parameters were measured? What were the populations metrics (means, standard deviations, etc) of the traits tested, what was the relationship between passive and active tameness measures (and indeed all measurements taken)? Similarly, what were the p-values for the tests between the strains, differences between strains in terms of standard deviations, etc.

Response: Sorry for this unclear description. We added some detailed information in measuring passive and active tameness (line 652-681). Active tameness was defined as animal actively approaching/contacting a human hand; and the passive tameness as the tolerance of the animal to the touch from human hand (See ref. 57 in the revised version). In this study, we used the active contacting time and passive tolerance time to measure the active and passive tameness. We added a table in summarizing the metrics of the tests in the supplementary materials (Additional file 1: Table S21).

9. The English language is good, but there are quite a few incidents of grammatical errors that pop up fairly often. I should stress that these are not so severe that they really disrupt the flow of reading, but they would certainly be good to address. For example (not exhaustive),
line 563 "to avoid hurts of the mouse", Corrected (line 659-660)
line 114 "were the world-widely used animal model", Corrected (line 113)
line 354 "Approximately 30% the male mutant individuals". Corrected (line 362-363)

Response: We asked a native English speaker to polish the whole manuscript. We modified the words or sentences as suggested.


Others:

We moved a paragraph of previous studies on Astn2 gene from results into the Discussion.

**Second round of review**

**Reviewer 1**

Although the authors have addressed most of my comments, I still don’t agree with the criteria used to reduce the number of SNPs most likely having been under selection. (lines 334-343).
(1) By making it mandatory that there are 3 alleles at a given SNP position, one selects for sites that are, on average, are more likely to be tolerant for nucleotide variations. On the other hand, rare variants at sites that are important for biological function, are more likely to be present only as bi-allelic SNPs. Such variants could be “deleterious” in wild animals because they reduce fitness. However, such a variant but might be strongly selected in domesticated animals. This criterium therefore is very likely to exclude many potential interesting variants that have been under selection.
(2) The reasoning in criterium 2 that “since a single allele in a classical inbred population may be caused by genetic drift or founder effect” is not a valid criterium. While I agree that this indeed very often will be the case, variants that are under strong selection during the inbreeding process also likely will become fixed.
In my opinion, these two criteria do NOT exclude or alleviate the interference of genetic drift or founder effects but reduce any kind of variant, also those that potentially have been under selection. Apparently, the authors were lucky in retaining and identifying the Asn mutation, but at the same time it is very likely that they have excluded many other potentially interesting mutations.

**Reviewer 2**

The authors have addressed all my points and I am satisfied with their reponses.

**Authors’ response to reviewers**

Reviewer #1:
Although the authors have addressed most of my comments, I still don't agree with the criteria used to reduce the number of SNPs most likely having been under selection. (lines 334-343).
(1) By making it mandatory that there are 3 alleles at a given SNP position, one selects for sites that are, on average, are more likely to be tolerant for nucleotide variations. On the other hand, rare variants at sites that are important for biological function, are more likely to be present only as bi-allelic SNPs. Such variants could be "deleterious" in wild animals because they reduce fitness. However, such a variant but might be strongly selected in domesticated animals. This criterium therefore is very likely to exclude many potential interesting variants that have been under selection.
(2) The reasoning in criterium 2 that "since a single allele in a classical inbred population may be caused by genetic drift or founder effect" is not a valid criterium. While I agree that this indeed very often will be the case, variants that are under strong selection during the inbreeding process also likely will become fixed.
In my opinion, these two criteria do NOT exclude or alleviate the interference of genetic drift or founder effects but reduce any kind of variant, also those that potentially have been under selection. Apparently, the authors were lucky in retaining and identifying the Asn mutation, but at the same time it is very likely that they have excluded many other potentially interesting mutations.

Response:
We sincerely apologize for these incorrect statements pointed out by the reviewer. We agree that criterion 1 and 2 do NOT exclude or alleviate the interference of genetic drift or founder effects, and could reduce potential interesting SNPs under selection. After further check, we now solved the problems in identifying the SNPs which were used for gene editing and behavioral tests.
As we have demonstrated in the Methods, at first, we performed an analysis to select genes by using wild mice of two subspecies M. m. domesticus and M. m. musculus, without using samples of wild subspecies M. m. castaneus. We found eight genes that exhibited a close relationship to mental illness and behavior, including Prkcq, Astn2, Gm20388, Pcdh15, Eea1, Nav3, Nrxn3, and Iglc2. We selected the sites for gene editing and behavioral tests using a simple rule that reference allele homozygous does not exist in any wild mice (n=21), but exists in all the classical inbred mice (n=28). For example, for an SNP with bi-allele (e.g. A,T, and T is the reference allele), if no individual of wild mice is TT (they may have AT, AA), but all individuals of classical inbred mice are TT, we conclude that this SNP would have been selected by domestication (although we can not exclude the effects of founder effect or genetic drift). Similarly, for an SNP with tri-allele (e.g., A,T,C, and T is the reference allele), if no individual of wild mice is TT (they may have AT, AC, TC, AA, CC), but all individuals of classical inbred mice are TT, we conclude that this SNP would have been selected by domestication. By using the rule, a total of 32 sites were found, including 4 sites in Astn2, 1 site in Gm20388, 1 site in Eea1, 3 sites in Nav3, and 23 sites in Nrxn3 (see Additional File 2: Table S22). After further filtering by considering the gene editing difficulty and excluding previous tested sites, a total of four SNPs was used for constructing the mouse model (here, we corrected a mistake in the previous version of Additional File 1: Table S23). It is notable that gene editing and behavioral tests have been completed before we conducted the secondary analysis using three subspecies of wild mice. Behavioral tests demonstrated that the SNP located at Chr4: 66226438 showed a significant difference in behavioral performance between wild and mutated types (See Fig. 4). We have added the description about the original rule in selecting the SNPs for constructing mouse models in Methods section (line 607-614; 620-627).
Later on, we succeeded in capturing wild mice of subspecies M. m. castaneus. Then, we re-ran the selection process by including three wild subspecies of M. m. domesticus, M. m. musculus and M. m. castaneus. We found only Astn2, Pcdh15, and Nrxn3 remained in the top selected genes. However, we were not able to select the SNP of Astn2 located at Chr4: 66226438 using the original rule. Of this SNP, the frequency of reference allele T was 0.0417 in wild mice, and 0.793 in inbred mice. Further analysis using the first part of the rule, that is, an SNP with no reference allele homozygous in all wild mice, showed that the SNP located at Chr4: 66226438 was included in the selected 69 SNPs of Astn2 (line 620-627; Additional File 2: Table S24). This suggest that the original rule may be extreme in filtering the potential selected SNPs under domestication. Therefore, in the previous revised version, we developed three criteria (criterion1, 2 and 3) to select the potential interesting SNPs. The principle of criterion 3 is similar to the original one, but with a much more relaxed condition than the original rule: the frequency of the reference allele in the wild mice is less than 20% of the frequency in classical inbred mice.
Using criterion 3, we obtained 196 SNPs (Additional File 2: Table S25) from in total 11,017 SNPs located at Astn2. Among the 196 SNPs, 7 SNPs are tri-allelic and 189 SNPs are bi-allelic in wild mice; 1 SNPs is tri-allelic, 161 SNPs are bi-allelic and 34 SNPs are with single allele in classical inbred mice (line 627-637; Additional File 1: Fig. S17). To further narrow down the number of potentially interesting SNPs, we used criterion 1 (only choosing multiple-allele SNPs in wild mice) and criterion 2 (only choosing bi-allele in inbred mice), which reduces the number of 196 to 5 SNPs. The SNP of Chr4: 66226438 is located in tri-allele in wild mice and bi-allele in classical inbred mice. Indeed, as pointed out by you, this strategy excluded too many potential SNPs under selection.
We agree the comments raised by you that, criteria 1 and 2 are unreasonable for SNP filtering. The two criteria would exclude many potentially interesting SNPs, and are not able to remove interference of founder effect and genetic drift. In this revised version, we deleted the relevant descriptions on criterion 1 and 2 (line 327-337), kept the description of criterion 3, and added Table S25 and revised Fig. S17 showing the list and their properties of the 196 SNPs filtered by criterion 3 (line 627-637). This part was moved to Method section.

**Third round of review**

**Reviewer 1**

In the new version you sufficiently have addressed my previous comments. I have no further comments.
